# Multimodal MRI-based radiomics in an ASD rat model: investigating brain structural changes and the neuroprotective effects of selenium

**DOI:** 10.3389/fnins.2025.1651220

**Published:** 2025-10-08

**Authors:** Yikai Shu, Xiaoan Zhang, Jun Huang, Chengdong Li, Yang Zhang

**Affiliations:** ^1^The First Affiliated Hospital, and College of Clinical Medicine of Henan University of Science and Technology, Luoyang, China; ^2^Henan University of Science and Technology, Luoyang, China; ^3^The Third Affiliated Hospital of Zhengzhou University (Henan Maternal and Child Health Hospital), Zhengzhou, China; ^4^The Second Affiliated Hospital of Henan University of Science and Technology, Luoyang, China; ^5^The First Affiliated Hospital of Baotou Medical College, Baotou, China

**Keywords:** autism, rats, selenium, magnetic resonance imaging, radiomics

## Abstract

**Introduction:**

This study developed and validated a multimodal MRI-based radiomics model to assess brain changes in a rat model of autism spectrum disorder (ASD) following selenium intervention.

**Methods:**

ASD was induced in Sprague–Dawley rats via prenatal valproic acid administration, with sodium selenite used for intervention. MRI modalities included T2-weighted imaging, T1 and T2 relaxation mapping, diffusion tensor imaging, and diffusion kurtosis imaging. Radiomics features were extracted, correlated with behavioral metrics, and analyzed using clustering and radiomics scoring. Logistic regression models incorporating single-modality and multimodal radiomics features were developed and evaluated using receiver operating characteristic (ROC) curve analysis. Subgroup analyses assessed predictive performance and correlations with behavioral and developmental indices.

**Results:**

ASD model rats exhibited growth retardation, anxiety-like behavior, and deficits in social interaction and memory, which were alleviated by selenium supplementation. The multimodal radiomics model outperformed single-modality models, achieving the highest area under the ROC curve and strong predictive capability in subgroup analyses. Significant correlations were identified between multimodal radiomics scores and behavioral as well as developmental measures.

**Discussion:**

The cerebellum was a key region affected in ASD, whereas the visual–auditory cortex showed notable responses to selenium treatment. In conclusion, the multimodal radiomics model demonstrates high diagnostic efficacy, highlights the cerebellum as a key region affected in ASD, and suggests the visual–auditory cortex as a primary target of selenium intervention, enhancing predictive accuracy for structural and functional brain improvements post-treatment.

## 1 Introduction

Autism spectrum disorder (ASD) is a genetic neurodevelopmental disorder characterized by impairments in social functioning, language communication, and repetitive behaviors (Lai et al., 2014), often accompanied by deficits in attention and cognitive functions (Volkmar et al., 2004). The global prevalence of ASD is approximately 1 in 44 (Malwane et al., 2022), with an estimated rate of 0.70% in China (Hai and Leng, 2022), and this rate has been steadily increasing in recent years (Aung et al., 2022; Jomova et al., 2022). While the exact pathogenesis of ASD remains unclear, accumulating evidence suggests that genetic, environmental, and immune factors all contribute to its development. Environmental influences, particularly those involving physical and chemical agents (Styles et al., 2020), have recently received increased scrutiny. These adverse factors may influence the development and function of the nervous system in individuals with ASD. Trace elements, such as selenium, play a critical role in nervous system development and functioning (Mlinarič et al., 2024). This raises the question of whether there is an association between selenium levels and ASD.

A cohort study found elevated plasma levels of heavy metals, such as chromium (Cr) and vanadium (V) (Zhang et al., 2022), in children with ASD. These high concentrations can activate DNA methylation sites, potentially affecting neurodevelopment (Leiter et al., 2022). Additionally, a comparison of ASD children with varying degrees of severity indicated that reduced selenium levels might correlate with structural brain changes and disorder (Wirth et al., 2014) severity. Our earlier population study also noted decreased serum selenium levels in children with ASD, suggesting that selenium deficiency may impair critical physiological functions, such as its role in mediating hippocampal neuron formation and maintaining learning and memory functions (Lambin et al., 2017). However, the precise role of selenium in ASD has yet to be fully elucidated. Therefore, based on the findings of the aforementioned population studies, there appears to be a potential relationship between selenium and ASD. To address the current gap regarding the role of selenium in the ASD brain, we aim to investigate its association with both the structure and function of the ASD brain, employing imaging-based approaches.

Before investigating the role of selenium in the ASD brain using imaging techniques, it is important to first clarify the applications of imaging in the diagnosis and management of ASD. Although no universal consensus exists on the specific neuroimaging characteristics of ASD presently, the use of quantitative magnetic resonance imaging (MRI) has provided valuable structural and functional insights into the neurophysiology of ASD and the developmental changes occurring in the autistic brain across childhood, adolescence, and adulthood (Rafiee et al., 2022). Therefore, our animal experiments will utilize multimodal MRI scanning to enhance our understanding of brain changes following selenium treatment and address the limitations of single-sequence MRI. This approach offers a comprehensive, multidimensional view of the brain, complemented by analysis using radiomics techniques. Radiomics involves extracting and quantifying texture features from medical images (Singh et al., 2023), capturing the spatial relationships between signals within the image (Havdahl et al., 2022). This facilitates the extraction of pathophysiological information from regions of interest (ROIs) and supports the development of disease biomarkers. Additionally, radiomics can aid in assessing disease progression and prognosis (Viana et al., 2023).

In order to clearly explore the role of selenium in the brain of ASD, we propose to explore the following scientific questions: Is there a relationship between selenium and the structural changes observed in the brains of ASD? Does selenium supplementation improve these structural and functional abnormalities? To investigate these questions, we will utilize an animal model for exploratory studies and propose the following preliminary hypothesis: Selenium deficiency in ASD may induce structural changes in the brain, leading to behavioral abnormalities. Conversely, selenium supplementation may target specific brain regions to mitigate these structural changes and associated behavioral abnormalities. To elucidate the effects of selenium treatment on brain structure and function, we will develop a multimodal neuroimaging-based radiomics model to investigate the role of selenium in brain structure and its potential mechanisms in ASD. This approach aims to provide a comprehensive understanding of therapeutic effects of selenium supplementation in ASD, which may offer potential benefits for the early diagnosis and treatment of individuals with ASD in the future.

## 2 Materials and methods

### 2.1 Animals and grouping

Sixteen to eighteen-week-old SPF-grade Sprague–Dawley rats (10 males and 20 females) were acquired from Beijing Vital River Laboratory Animal Technology Co., Ltd. (License No. SCXK-2021-0011) and housed in gender-segregated cages. The rats were maintained under a controlled 12-h light/dark cycle, with unrestricted access to food and water. All experimental procedures were carried out between 10:00 a.m. and 3:00 p.m. and received approval from the Institutional Ethics Committee (Ethics No.: 2023–340-01).

### 2.2 Modeling

Male and female rats were co-housed in a 1:2 ratio overnight at 7:00 p.m. The following morning at 7:00 a.m., males were removed, and vaginal smears were collected from the females. The presence of sperm confirmed the onset of pregnancy at 0.5 days. On gestational day 12.5, pregnant females received an intraperitoneal injection of 50 mg/mL valproic acid (VPA) sodium salt (Biological Engineering Co., Ltd., Shanghai, A604040) at 600 mg/kg. Offspring from these females formed the VPA group, with postnatal day 1 (PN1) marked as the birth date. A separate cohort of healthy pregnant females was administered a saline solution intraperitoneally as a control, with their offspring forming the control group.

On postnatal day 28, the VPA and control groups were subdivided into two additional groups, each resulting in four experimental groups: VPA, VPA + Se, control, and control+Se. The selenium-supplemented groups received 1 mg/kg/day of sodium selenite solution (Sigma, 71,950) in their drinking water for 4 weeks, whereas the other groups received standard drinking water. Each group consisted of eight male rats, and developmental and behavioral assessments were conducted three times. All developmental and behavioral data from three independent replicates will be used to construct the radiomics model.

### 2.3 Developmental assessment

Developmental indicators were assessed at multiple points. Body weight was measured on postnatal days 1, 3, 5, 7, 14, 21, 30, 35, 42, 49, and 60 to track growth trajectories. Tail length was measured on postnatal days 1, 3, 5, 7, 14, 21, and 30 as an additional growth marker. Eye-opening was monitored from postnatal days 13 to 16, scoring as follows: 0 if neither eye was open, 1 if one eye was open, and 2 if both eyes were open.

Reflex development, balance coordination, and brain maturation were evaluated using the negative geotaxis test between postnatal days 7 and 10. Each pup was placed head-down on a 30-degree inclined acrylic board, and the time taken to rotate 180° to a head-up position was recorded. Motor function development was assessed via swimming tests on postnatal days 8, 10, 12, and 16 in a 28–29 °C water tank. Swimming posture was observed and scored with criteria as follows: 0 if both nose and head were submerged; 1 if only the nose was submerged; 2 if both nose and top of the head were at or above the water surface while ears remained submerged; 3 if ears were partially above the water; and 4 if ears were entirely above the water surface.

### 2.4 Behavioral assessment

Behavioral experiments were conducted using the ANY-Maze animal behavior video tracking software (Stoelting, USA), with the experimental equipment and chambers supplied by Grobel Company (China).

### 2.5 Open field test

The open field chamber, measuring 60 × 60 × 60 cm (Grobel, GB-OPA), was divided into 25 equal squares, designating the central nine squares as the central zone. Rats were acclimated to the testing room one day prior to the experiment. During testing, each rat was positioned consistently facing the wall at the start and allowed to roam freely for 10 min. The ANY-Maze system automatically recorded each rat’s time in the central zone and calculated the ratio of central zone time to the total time to evaluate spontaneous behavior and anxiety levels. The test area was maintained in quiet conditions, and the chamber was sanitized with 75% ethanol after each session.

### 2.6 Buried object test

A 60 × 30 × 30 cm box (Grobel, GB-OPA) with a 5 cm layer of wood bedding was utilized. Twenty black glass marbles, each 1 cm in diameter, were arranged in a 4 × 5 grid on the bedding. Rats had an acclimation period before testing commenced. During the test, rats could move freely for 30 min, and marbles buried by more than 50% of their volume were counted to measure anxiety levels. The bedding was replaced fresh for each animal to ensure consistent test conditions.

### 2.7 Three-chamber social test

The apparatus was a black box (60 × 40 × 20 cm, Grobel, SA219R) divided into three interconnected sections by two transparent movable panels. The test was structured in three 10-min phases. In the first phase, the rat explored an empty apparatus. During the second phase, an unfamiliar same-sex, same-age rat was introduced to one side chamber, leaving the other empty. In the final phase, another unfamiliar rat was placed in the previously empty chamber, allowing the test rat to explore all sections freely. The time and frequency of interactions with the new rats were documented to assess social behavior and preferences. The environment was kept quiet, and the apparatus was cleaned with 75% ethanol after each test.

### 2.8 Novel object recognition test

The experimental setup involved an empty 60 × 60 × 60 cm box (Grobel, GB-OPA). Before the tests, rats were acclimatized by placing them in the box. During the training phase, two identical objects were introduced into the box, allowing the rats to freely explore for 10 min while their interactions with the objects were documented. The testing phase occurred one hour later, substituting one of the previously introduced objects with a new one. The rats were then allowed another 10-min exploration period. Exploration behaviors toward both the new and familiar objects were recorded to evaluate the rats’ memory and learning capabilities. The recognition index (RI) was calculated using the formula: RI = (Time spent exploring the new object)/(Time spent exploring the new object + Time spent exploring the old object).

### 2.9 Magnetic resonance imaging

MRI scans were performed using a 4.7 T MR Solution small animal MRI system (MR Solution, UK). Rats were deeply anesthetized using an isoflurane anesthesia machine (Shenzhen Revo Life Science Technology Co., Ltd.) and subsequently positioned on the scanning bed in a prone orientation, aligning the head along the sagittal plane and the body with the axis of the bed. The head was centered within the magnetic field of the head coil, and all necessary connections were made. Scanning protocols included Scout, T2, T1 Mapping, T2 Mapping, diffusion tensor imaging (DTI), and diffusion kurtosis imaging (DKI). Images exhibiting significant artifacts were discarded, and the remaining data were processed post-scan. MRI scans were completed for all rats undergoing three developmental and behavioral testing cycles. A total of 20 imaging datasets were sequentially collected in the CON group, CON+Se, and ASD group, whereas 22 datasets were collected in the ASD + Se group. Fourteen rats were excluded from further analysis for the following reasons: 4 rats from the CON group, 4 from the CON+Se group, 4 from the ASD group, and 2 from the ASD + Se group. The exclusion criteria were: (1) absence of diffusion tensor imaging or diffusion kurtosis imaging (2 rats in the ASD + Se group); (2) poor image quality due to incomplete images or high levels of artifacts (4 rats in the CON+Se group and 4 in the ASD group); (3) excessive head motion (4 rats in the CON group excluded). All remaining datasets were used for the subsequent construction of the radiomics models.

### 2.10 Pre-processing

All images were analyzed using in-house developed tools and Python software (version 3.7.4). Data pre-processing included visual inspection of images for quality assessment and removal of artefactual images; image smoothing; correction for motion and eddy current distortions; correction for distortions caused by B0 field inhomogeneities using the acquired field map data and tools; adjustment of the encoding gradient matrix to account for rotations during motion correction; and brain tissue extraction and segmentation of regions of interest (ROI) from structural images.

Following image correction, T2-weighted imaging and DKI data were exported in DICOM format and imported into ITK-SNAP software (version 4.0.1, Image Computing and Science Laboratory, University of Pennsylvania). Regions of interest (ROIs) were delineated semi-automatically using Python software and manually refined to facilitate the acquisition of multiple brain regions, including the frontal lobe, visual cortex, motor cortex, sensory cortex, auditory cortex, cingulate gyrus, corpus callosum, internal capsule, hippocampus, thalamus, cerebellum, and amygdala. Mean values, voxel counts, and coordinates were meticulously extracted and recorded for each ROI (Paxinos and Watson, 1997, 2005).

MRI data for T1 and T2 mapping were exported in NIfTI format following correction. These datasets were then imported into T1 mapping software (version 3.1, MR Solution, UK) and T2 mapping software (version 3.2, MR Solution, UK). After data processing, pseudo-color maps were generated for both T1 and T2 sequences, visually representing signal intensities with distinct colors. ROIs were automatically outlined on these maps to compute mean T1 and T2 values.

DTI and DKI data were also exported in NIfTI format following correction and pre-processing and processed using DTI Studio software (version 2.0, Johns Hopkins University). The post-processing workflow encompassed the generation of b-value tables, gradient tables, and reconstructed brain masks. Parameter maps for fractional anisotropy, mean diffusivity, radial diffusivity, and axial diffusivity were created alongside tractography images that depicted neural fiber pathways within the brain. This software facilitated the alignment of ROIs with corresponding DTI, DKI, and parameter values, ensuring precise data analysis and interpretation.

### 2.11 Radiomics processing

#### 2.11.1 Radiomics feature extraction

Radiomics features were extracted from ten manually delineated regions on T2-weighted MR images using PyRadiomics on the Python platform.

#### 2.11.2 Feature selection

The Pearson correlation coefficient (r) was calculated between all radiomics features derived from T2, DTI, DKI, T1-, and T2-mapping sequences, as well as the corresponding behavioral features. Results were transformed into F-scores, and *p*-values were computed. Multiple testing correction was applied using the Benjamini/Hochberg method, setting a threshold of 0.05 to identify significantly correlated radiomics features. These significant features were then combined to form the final selected feature set. Clustering analysis was conducted on these features to eliminate redundancy due to high correlation.

#### 2.11.3 T2 radiomics signature construction

A unique T2 radiomics signature was developed for each delineated region. Initially, clustering analysis was performed on the test rats, utilizing the results as targets for constructing the radiomics signature model. This clustering was executed using the Agglomerative Clustering method.

#### 2.11.4 Construction of the radiomics score (RadScore)

Univariate logistic regression analysis was performed on the selected measurement features and radiomics signatures for both the modeling and treatment prediction targets. Features with a *p*-value below 0.05 were advanced to multivariate logistic regression for model development. If no features met the p-value threshold, the feature with the smallest p-value was selected for the final model, referred to as the Radiomics Score (RadScore). Model comparison and sensitivity analysis were performed to evaluate the model, with the Radiomics Score fully utilized as a key evaluation metric.

### 2.12 Statistical analysis

Data were organized using Excel and analyzed using SPSS 24.0. All data visualization and further analysis were conducted in Prism 10.0 (GraphPad Software). Results are reported as mean ± standard deviation (SD). Differences in developmental indicators were assessed using an unpaired Student’s t-test. For eye-opening and swimming scores, the Mann–Whitney U test was applied. Significant findings from one-way ANOVA prompted individual comparisons using the Tukey *post hoc* test. Similarly, significant results from Kruskal-Wallis tests led to individual comparisons using Dunn’s test. Pearson correlation analysis was employed to evaluate the correlation between two independent samples. For multiple comparisons, the Benjamini/Hochberg correction method was used. Clustering analysis utilized the Agglomerative Clustering method, employing Euclidean distance for each sample, ward linkage for inter-cluster distance calculation, and Davies-Bouldin score optimization for distance threshold determination. Receiver operating characteristic (ROC) curve analysis was performed for both single-modality and multi-modality imaging scores to predict outcomes in modeling and treatment scenarios. The DeLong test was employed to compare differences in AUC between the imaging scores. Statistical significance was defined as a *p*-value of less than 0.05 for all comparisons.

## 3 Results

### 3.1 Se supplementation partially improved growth retardation in ASD rats

**Figure 1:**
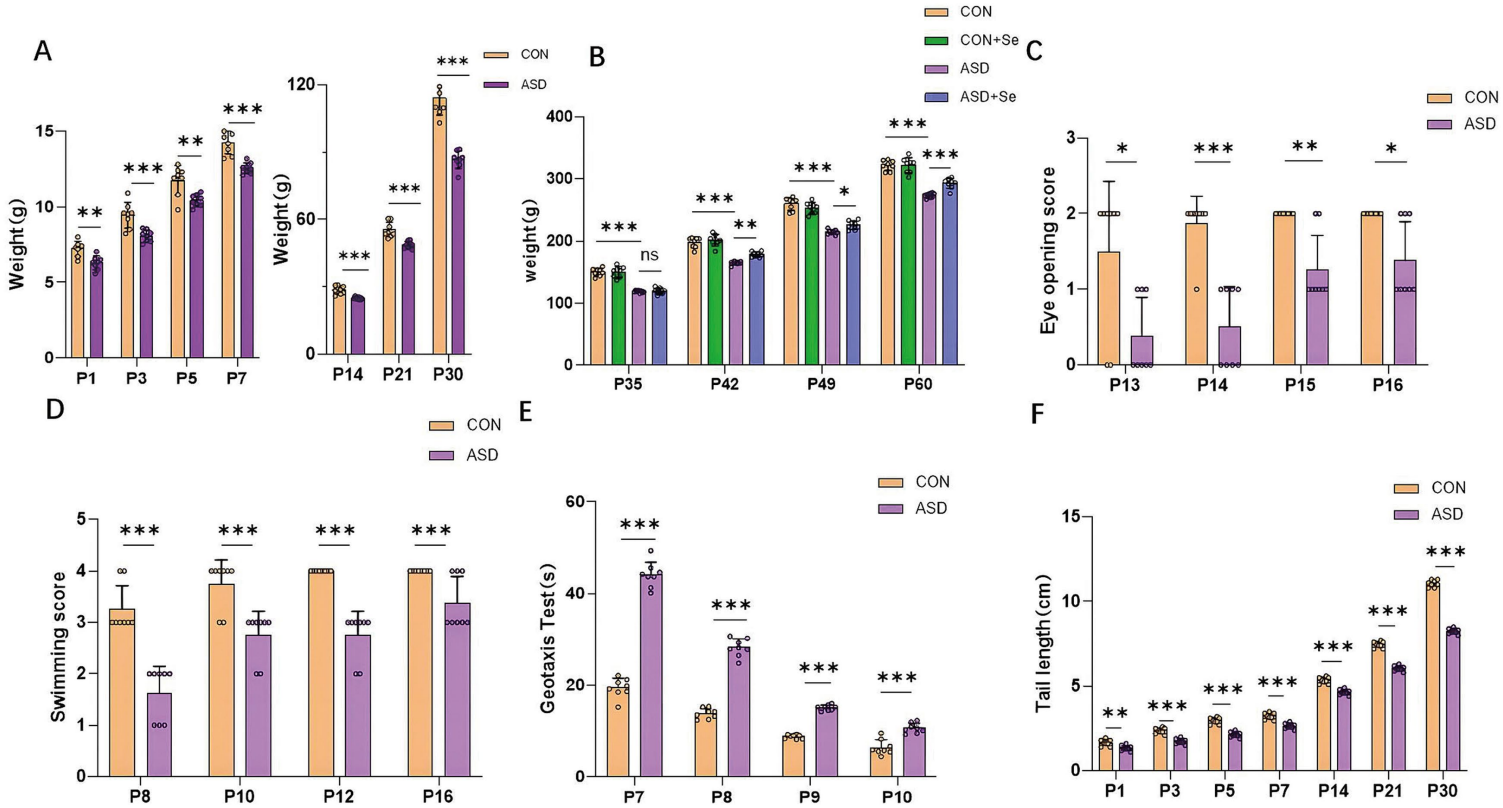
Developmental results: **(A)** Comparison of body weight between CON and ASD groups from P1 to P30. **(B)** Body weight comparison between CON, CON+Se, ASD, and ASD + Se groups at P35, P42, P49, and P60. **(C)** Eye-opening developmental scores in the CON and ASD groups. **(D)** Swimming scores in the CON and ASD groups. **(E)** Comparison of negative geotaxis time between CON and ASD groups. **(F)** Comparison of tail length between CON and ASD groups. All data are presented as mean ± SD (*n* = 8). ns, not significant; ^*^p < 0.05; ^**^p < 0.01; ^***^p < 0.001.

We found that the body weight at P1, P3, P5, P7, P14, P21, and P30 was significantly higher in the CON group compared to the ASD group [Figure 1A, *n* = 8 rats/group, ^**^p < 0.01, ^***^p < 0.001; unpaired *t* test; P1: *t*_(14)_ = 3.97, *p* = 0.001; P3: *t*_(14)_ = 4.17, *p* < 0.001; P5: *t*_(14)_ = 3.52, *p* = 0.003; P7: *t*_(14)_ = 5.74, *p* < 0.001; P14: *t*_(14)_ = 5.39, *p* < 0.001; P21: *t*_(14)_ = 5.06, *p* < 0.001; P30: *t*_(14)_ = 9.22, *p* < 0.001]. Furthermore, we compared the weight changes after selenium treatment across four groups (CON, CON+Se, ASD, and ASD + Se) at P35, P42, P49, and P60. Statistical analysis revealed significant differences among the four groups [Figure 1B, *n* = 8 rats/group, ns: not significant,^*^p < 0.05, ^**^p < 0.01, ^***^p < 0.001; one-way ANOVA comparing the four groups at P35, *F*_(3, 28)_ = 74.25, *p* < 0.001, one-way ANOVA with Tukey’s *post hoc*: CON verse ASD, *p* < 0.001; ASD verse ASD + Se, *p* = 0.9975; one-way ANOVA comparing the four groups at P42, *F*_(3, 28)_ = 52.46, *p* < 0.001, one-way ANOVA with Tukey’s post hoc: CON verse ASD, *p* < 0.001; ASD verse ASD + Se *p* = 0.002; one-way ANOVA comparing the four groups at P49, *F*_(3, 28)_ = 60.92, *p* < 0.001, one-way ANOVA with Tukey’s post hoc: CON verse ASD, *p* < 0.001; ASD verse ASD + Se *p* = 0.041; one-way ANOVA comparing the four groups at P60, *F*_(3, 28)_ = 59.05, *p* < 0.001, one-way ANOVA with Tukey’s post hoc: CON verse ASD, *p* < 0.001; ASD verse ASD + Se, *p* = 0.0007]. The CON group showed significantly higher eye-opening scores at all observation points than the ASD group (Figure 1C, *n* = 8 rats/group, ^*^p < 0.05, ^**^p < 0.01, ^***^p < 0.001; Mann–Whitney U test between CON group and ASD group; postnatal day 13: *U* = 11, *p* = 0.027; postnatal day 14: *U* = 2, *p* < 0.001; postnatal day 15: *U* = 8, *p* = 0.007; postnatal day 16: *U* = 12, *p* = 0.025). Moreover, the CON group achieved significantly higher scores in swimming tests across all observation points than the ASD group (Figure 1D, *n* = 8 rats/group, ^***^p < 0.001; Mann–Whitney U test between CON group and ASD group; postnatal day 8: *U* = 0, *p* < 0.001; postnatal day 10: *U* = 6, *p* = 0.004; postnatal day 12: *U* = 0, *p* < 0.001; postnatal day 16: *U* = 12, *p* = 0.025). Similarly, the CON group demonstrated significantly shorter turning times than the ASD group at each observation time in the negative geotaxis test [Figure 1E, *n* = 8 rats/group, ^***^p < 0.001; unpaired *t* test between CON group and ASD group; postnatal day 7: *t*_(14)_ = 19.95, *p* < 0.001; postnatal day 8: *t*_(14)_ = 18.25, *p* < 0.001; postnatal day 9: *t*_(14)_ = 23.66, *p* < 0.001; postnatal day 10: *t*_(14)_ = 5.93, *p* < 0.001]. Lastly, the tail length of the CON group was significantly longer than that of the ASD group at each test point [Figure 1F, *n* = 8 rats/group, ^**^p < 0.01, ^***^p < 0.001; unpaired *t*-test between CON group and ASD group; postnatal day 1: *t*_(14)_ = 3.572, *p* = 0.003; postnatal day 3: *t*_(14)_ = 7.612, *p* < 0.001; postnatal day 5: *t*_(14)_ = 9.39, *p* < 0.001; postnatal day 7: *t*_(14)_ = 6.64, *p* < 0.001; postnatal day 14: *t*_(14)_ = 8.10, *p* < 0.001; postnatal day 21: *t*_(14)_ = 16.33, *p* < 0.001; postnatal day 30: *t*_(14)_ = 31.25, *p* < 0.001], indicating developmental delays in the ASD model rats.

### 3.2 Behavioral analysis

Based on developmental outcomes, we conducted behavioral tests on rats to determine the impact of selenium supplementation on their behavioral performance.

### 3.3 Se supplementation ameliorated spontaneous behaviors and anxiety in ASD rats

**Figure 2:**
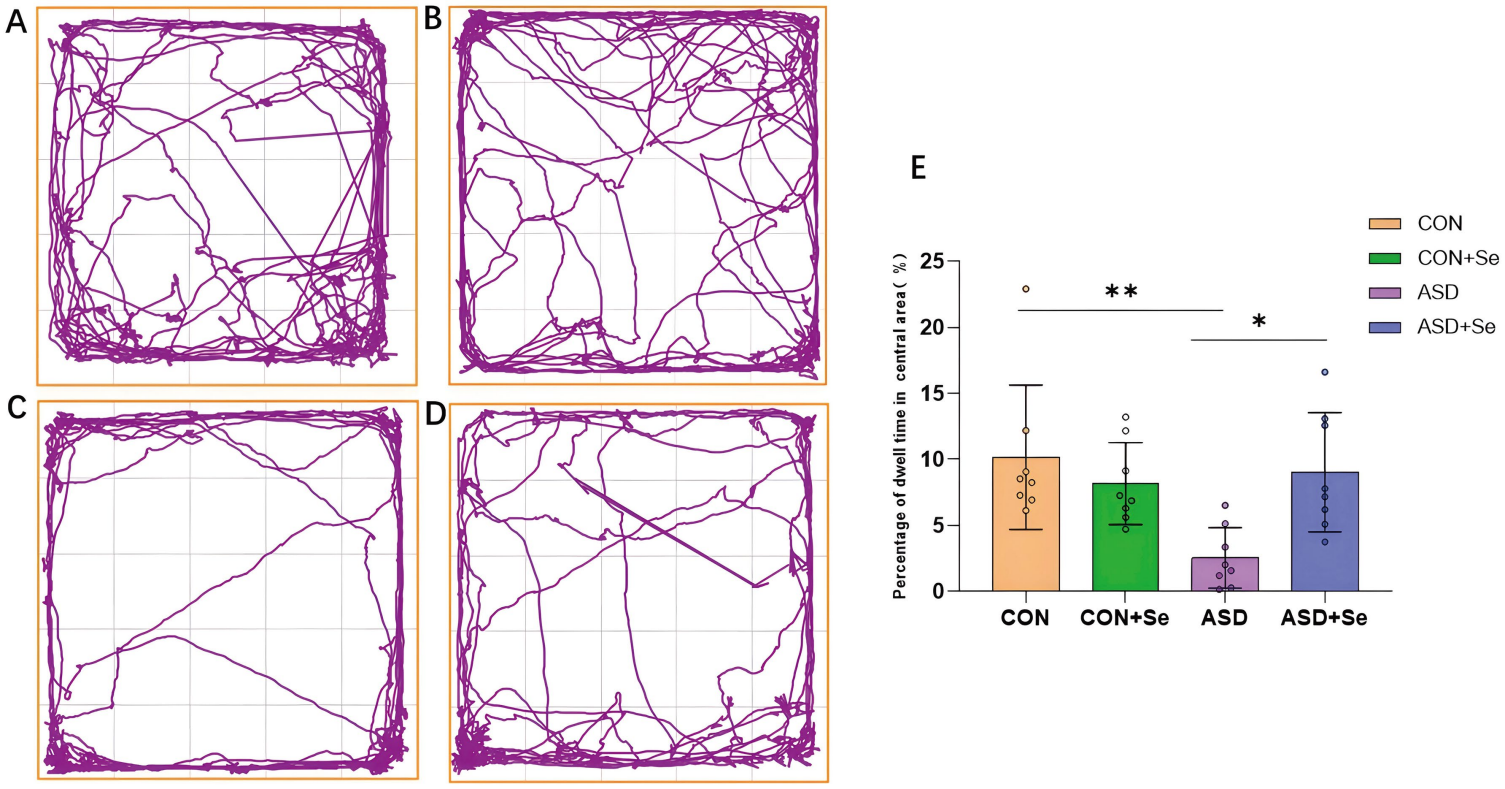
Effects of selenium supplementation on spontaneous exploration and anxiety: The proportion of time spent in the central area during the open field test is shown. **(A)** Movement tracks of the CON group. **(B)** Movement tracks of CON+Se group. **(C)** Movement tracks of the ASD group. **(D)** Movement tracks of the ASD + Se group. **(E)** Statistical results for each group. All data are presented as mean ± SD (*n* = 8). ns, not significant, ^*^p < 0.05; ^**^p < 0.01; ^***^p < 0.001.

To examine the effects of selenium supplementation on spontaneous behavior and anxiety levels, an open-field test was conducted. The findings indicated that rats in both the CON and CON+Se groups exhibited increased activity within the central area, characterized by more frequent entries and longer distances traveled, compared to other groups (Figures 2A–D). Notably, the CON group demonstrated significantly more time spent in the central area than the ASD group.

Moreover, after selenium treatment, rats in the ASD + Se group spent more time in the central area and covered a greater distance than the ASD group, with a significant difference in the percentage of time spent in the central area [Figure 2E, n = 8 rats/group, ^*^p < 0.05, ^**^p < 0.01, one-way ANOVA comparing the four groups, *F*_(3, 28)_ = 5.658, *p* = 0.0037, one-way ANOVA with Tukey’s *post hoc*: CON verse ASD, *p* = 0.004; ASD verse ASD + Se, *p* = 0.016].

### 3.4 Se supplementation ameliorated anxiety in ASD rats

**Figure 3:**
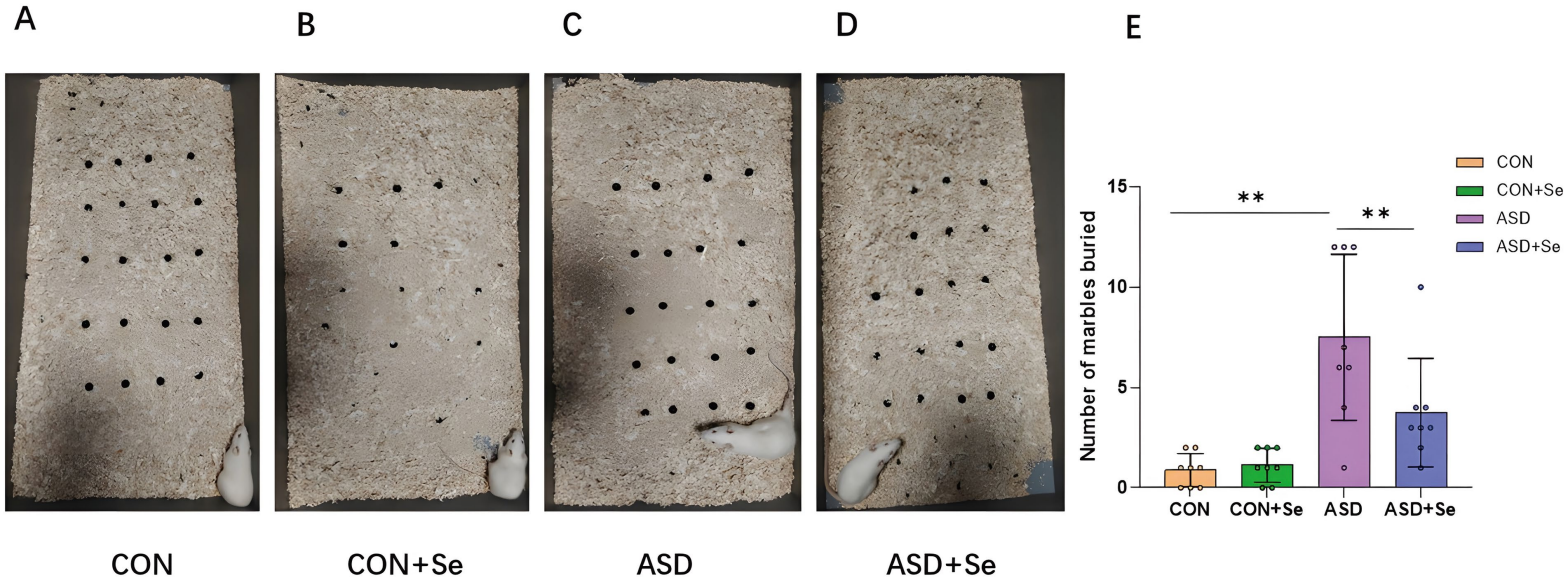
Effects of selenium supplementation on anxiety behaviors: The number of glass beads buried after testing was recorded. **(A)** Sample image of beads buried in CON group; **(B)** Sample image of beads buried in CON+Se group; **(C)** Sample image of beads buried in ASD group; **(D)** Sample image of beads buried in ASD + Se group; **(E)** statistical results in four groups. All data are presented as mean ± SD (*n* = 8). ns, not significant; ^*^p < 0.05; ^**^p < 0.01; ^***^p < 0.001.

To further explore the impact of selenium supplementation on anxiety, we assessed anxiety severity in each rat group by counting the number of buried marbles, a recognized indicator of anxiety. As depicted in Figures 3A–D, the ASD group buried significantly more marbles than all other groups. Notably, there was a significant difference in the number of buried marbles between the CON and ASD groups. After selenium supplementation, the ASD + Se group buried fewer marbles compared to the ASD group, indicating a significant reduction in anxiety-related behaviors (Figure 3E, *n* = 8 rats/group, ^**^p < 0.01; Kruskal-Wallis test comparing the four groups, *H*_3_ = 18.91, *p* = 0.0003, Kruskal-Wallis with Dunn post hoc: CON vs. ASD, *p* = 0.0013; ASD vs. ASD + Se, *p* = 0.015). These results suggest that selenium supplementation may alleviate repetitive and anxious behaviors in ASD rats.

### 3.5 Se supplementation ameliorated social behavior in ASD rats

**Figure 4:**
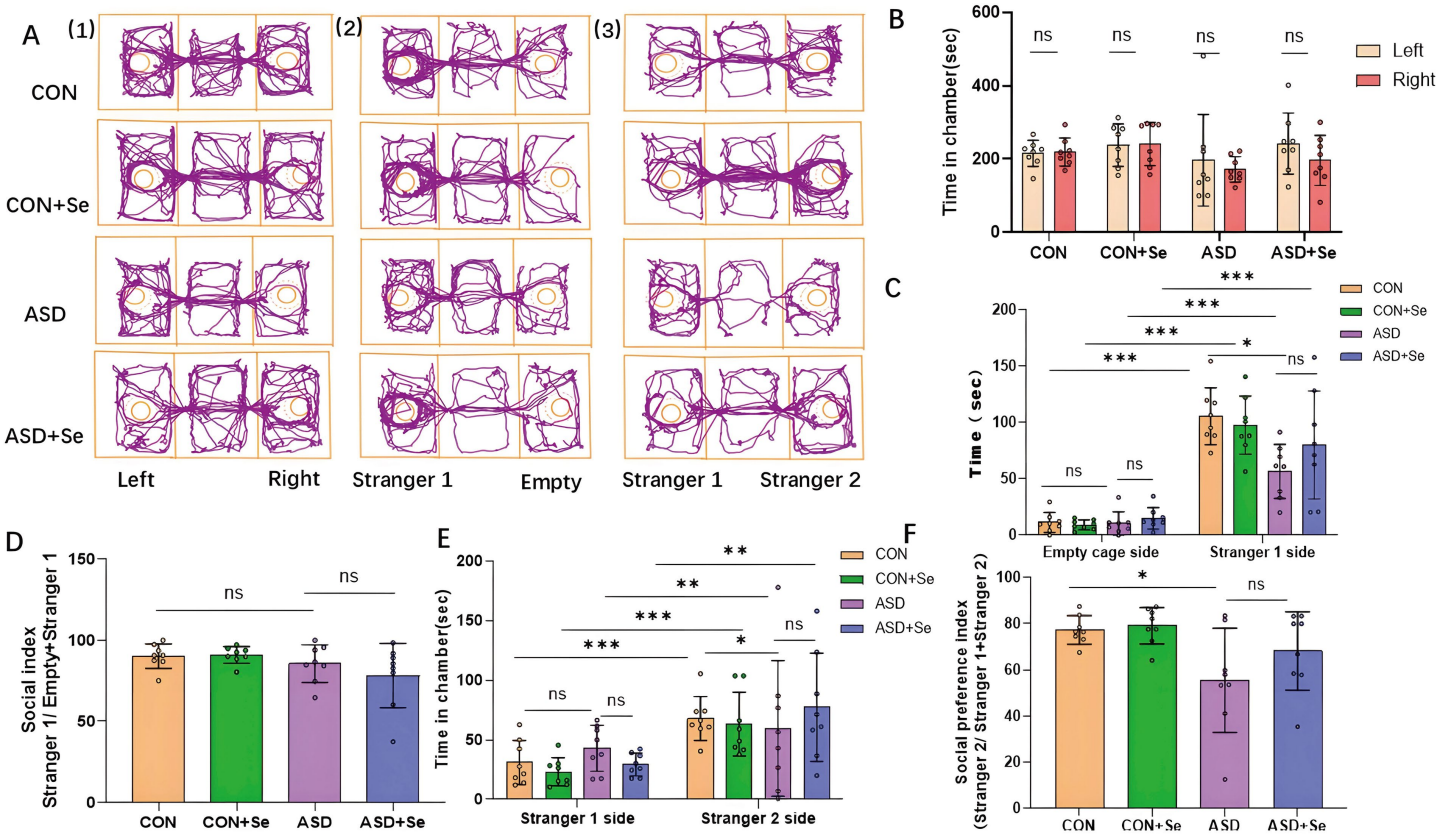
Effects of selenium supplementation on social behavior: Social ability and social preference depend on time spent. The social interaction index (SI) = (time spent on unfamiliar cage)/(time spent on unfamiliar cage + time spent on empty cage). The social preference index (SPI) = (time spent on unfamiliar cage) / (time spent on unfamiliar cage + time spent on familiar cage). **(A1)** Representative trajectories from the four groups during the habituation phase. **(A2)** Representative trajectories from the four groups during the social ability test. **(A3)** Representative trajectories from the four groups during the social preference test. **(B)** Statistical analyses of the habituation phase. **(C)** Statistical analyses of the social ability test. **(D)** Statistical analyses of the social index. **(E)** Statistical analyses of the social preference test. **(F)** Statistical analyses of the social preference index. All data are presented as mean ± SD (*n* = 8). ns, not significant; ^*^p < 0.05; ^**^p < 0.01; ^***^p < 0.001.

After assessing emotional and simple behavioral responses, we explored the social behavior of the experimental rats using the three-chamber social interaction test. During the exploration phase, no significant differences were observed in the time spent by rats in the left and right chambers across the four groups [Figures 4A-1,B *n* = 8 rats/group, ns: not significant, unpaired *t*-test comparing the left and right chambers: CON group: *t*_(14)_ = 0.198, *p* = 0.845; CON+Se group: *t*_(14)_ = 0.113, *p* = 0.911; ASD group: *t*_(14)_ = 0.548, *p* = 0.591; ASD + Se group: *t*_(14)_ = 1.205, *p* = 0.248], indicating no preference for either side of the chambers.

In the social interaction phase, all groups significantly preferred spending more time near Stranger 1 compared to the empty side [Figures 4A-2,C, *n* = 8 rats/group, ^***^p < 0.001, unpaired *t*-test between the empty chamber and Stranger 1 chamber: CON group: *t*_(14)_ = 9.965, *p* < 0.001; CON+Se group: *t*_(14)_ = 9.531, *p* < 0.001; ASD group: *t*_(14)_ = 5.002, *p* < 0.001; ASD + Se group: *t*_(14)_ = 3.759, *p* = 0.002], showing a general preference for social interaction. When analyzing the time spent on the empty side and the Stranger 1 side, no significant differences were found between the groups for time spent on the empty side [Figure 4C, *n* = 8 rats/group, ns: not significant, one-way ANOVA, *F*_(3, 28)_ = 0.668, *p* = 0.5785]. However, significant differences were observed on the Stranger 1 side.

The CON group demonstrated a significantly greater amount of time spent on the Stranger 1 side compared to the ASD group. Following selenium treatment, the ASD + Se group spent significantly more time on the Stranger 1 side compared to the ASD group. [Figure 4C, *n* = 8 rats/group, ns: not significant, ^*^p < 0.05; one-way ANOVA comparing the four groups, *F*_(3, 28)_ = 3.595, *p* = 0.0258, Tukey’s *post hoc*: CON vs. ASD, *p* = 0.0256; ASD vs. ASD + Se, *p* = 0.484]. Furthermore, analysis of social index scores revealed that the CON group exhibited higher social index values than the ASD group, although the differences were not statistically significant. Similarly, no significant difference was observed between the ASD and ASD + Se groups [Figure 4D, *n* = 8 rats/group; ns: not significant; one-way ANOVA, *F*_(3, 28)_ = 1.786, *p* = 0.1727].

### 3.6 Se supplementation ameliorated social preference based on social interaction

To assess social preference further, we analyzed the time spent by rats on the Stranger 2 side compared to the Stranger 1 side (Figure 4A-3). Results indicated that rats across all groups significantly favored the Stranger 2 side over Stranger 1 [Figure 4E, *n* = 8 rats/group, ^**^p < 0.01, ^***^p < 0.001, unpaired *t-*test between the stranger 1 chamber and stranger 2 chamber across the four groups. Stranger 1 vs. stranger 2 are presented here: in the CON group, *t*_(14)_ = 3.985, *p* < 0.001; in the CON+Se group, *t*_(14)_ = 4.112, *p* < 0.001; in the ASD group, *t*_(14)_ = 2.754, *p* = 0.01; in the ASD + Se group, *t*_(14)_ = 2.925, *p* = 0.01], suggesting a preference for Stranger 2. This finding implies that all experimental groups exhibited varying degrees of social preference. Subsequent statistical analysis of the time spent on the familiar revealed no significant differences among the four groups [Figure 4E, *n* = 8 rats/group, ns: not significant; one-way ANOVA comparing the four groups, *F*_(3, 28)_ = 1.103, *p* = 0.3644]. The ASD group, notably, spent significantly less time with the unfamiliar stranger compared to the CON group [Figure 4E, *n* = 8 rats/group, ns: not significant, ^*^p < 0.05; one-way ANOVA, *F*_(3, 28)_ = 3.871, *p* = 0.0196, Tukey’s *post hoc*: CON vs. ASD, *p* = 0.0185; ASD vs. ASD + Se, *p* = 0.1962]. Furthermore, the social preference scores were significantly lower in the ASD group compared to the CON group [Figure 4F, *n* = 8 rats/group, ns: not significant, ^*^p < 0.05; one-way ANOVA, *F*_(3, 28)_ = 4.179, *p* = 0.0145, Tukey’s *post hoc*: CON vs. ASD, *p* = 0.0329; ASD vs. ASD + Se, *p* = 0.3449].

### 3.7 Se supplementation ameliorated learning and memory in ASD rats

**Figure 5:**
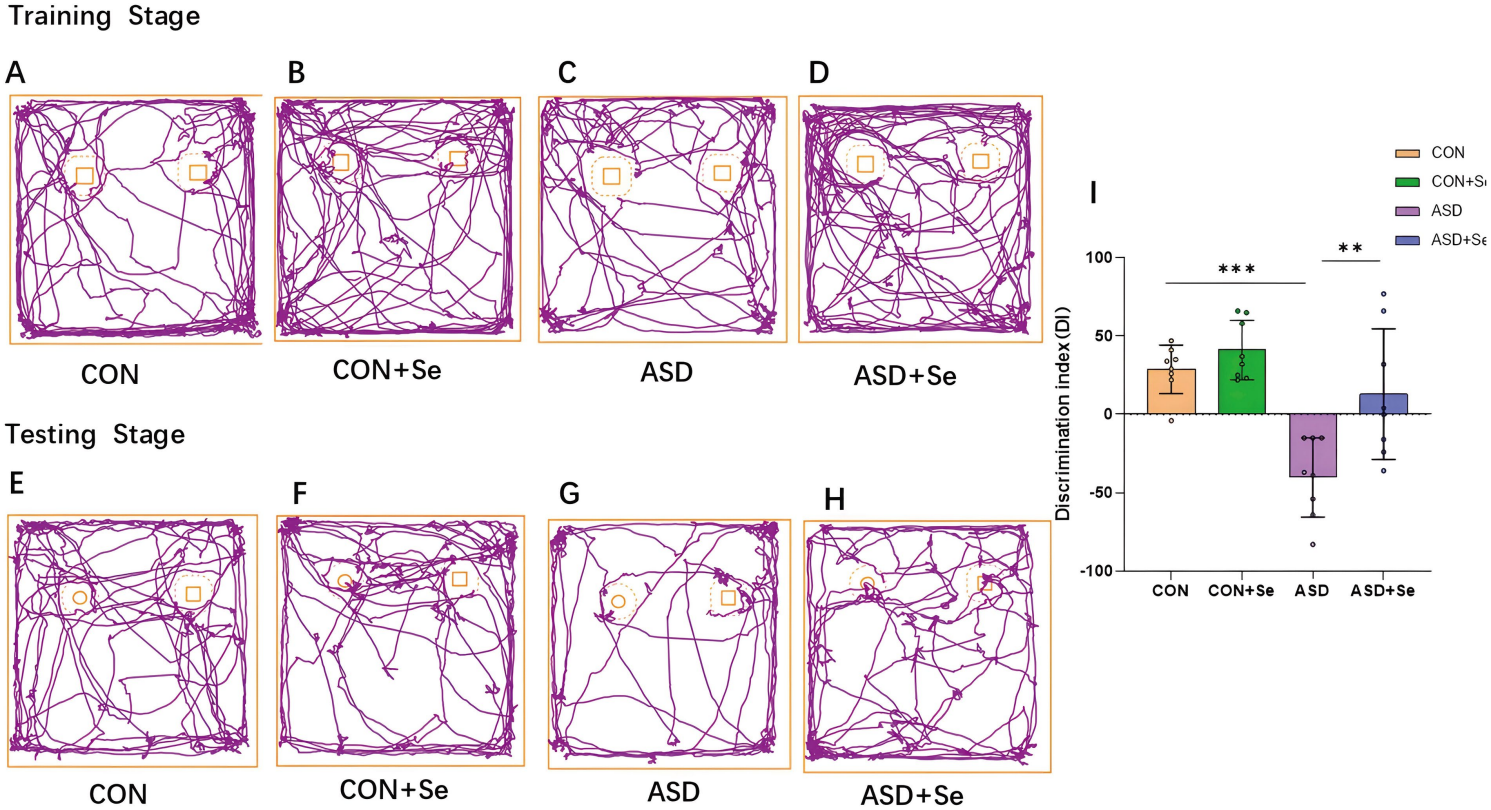
Effects of selenium supplementation on learning and memory: The time spent exploring an unfamiliar versus familiar object was recorded to calculate the DIs = (time spent with unfamiliar object - time spent with familiar object)/(time spent with unfamiliar object + time spent with familiar object). **(A)** Trajectory in the CON group during the learning training stage test (two identical objects). **(B)** Trajectory in the CON+Se group during the learning training stage test (two identical objects). **(C)** Trajectory in the ASD group during the learning training stage test (two identical objects). **(D)** Trajectory in the ASD + Se group during the learning training stage test (two identical objects). **(E)** Trajectory in the CON group during the memory test stage test (one unfamiliar and one familiar object). **(F)** Trajectory in the CON+Se group during the memory test stage test (one unfamiliar and one familiar object). **(G)** Trajectory in the ASD group during the memory test stage test (one unfamiliar and one familiar object). **(H)** Trajectory in the ASD + Se group during the memory test stage test (one unfamiliar and one familiar object). **(I)** Statistical results for the DIs. All data are presented as mean ± SD (*n* = 8). ns, not significant; ^*^p < 0.05; ^**^p < 0.01; ^***^p < 0.001.

In addition to evaluating social behavior, we examined the impact of selenium supplementation on higher cognitive functions, specifically learning and memory. During the learning and exploration phase, rats from all groups spent similar amounts of time interacting with two identical objects, showing no preference for either (Figures 5A–D). However, during the testing phase, the CON, CON+Se, and ASD + Se groups exhibited significantly more engagement with the novel object compared to the familiar one (Figures 5E–H). The discrimination index (DI) comparisons revealed that the DI of the CON group was significantly higher than that of the ASD group, and the DI of the ASD + Se group exceeded that of the ASD group following selenium intervention [Figure 5I, *n* = 8 rats/group, ^**^p < 0.01, ^***^p < 0.001, one-way ANOVA, *F*_(3, 28)_ = 13.78, *p* < 0.001, Tukey’s post hoc: CON vs. ASD, *p* < 0.001; ASD vs. ASD + Se, *p* = 0.003]. These findings suggest that selenium supplementation can enhance cognitive functions.

### 3.8 Radiomics analysis

After selecting the radiomic features extracted from each region, we found no behavior-related features in the amygdala, thalamus, or frontal lobe. Conversely, a single behaviorally relevant feature was identified for both the sensorimotor cortex and cerebellum (Supplementary Figures S1, S2), with multiple relevant features noted in other regions (Supplementary Figures S3a–S6b). Clustering analysis pinpointed predictive factors within the corpus callosum, internal capsule, hippocampus, and visual–auditory cortical regions (Supplementary Figures S7a–S10c).

#### 3.8.1 Unimodal models

Constructed unimodal models based on different sequences yielded diverse predictive factors related to modeling and treatment. For modeling prediction, the DTI sequence showed no significant predictive factors, whereas the DKI sequence revealed a predictive factor in the hippocampus (DKI-hippocampus-Volume). The T1 mapping sequence identified three predictive factors: mean value in the cerebellum (T1-mapping-cerebellum-mean), standard deviation in the internal capsule (T1-mapping-internal capsule-SD), and standard deviation in the hippocampus (T1-mapping-hippocampus-SD). The T2 mapping sequence highlighted two predictive factors: mean pixel intensity in the cerebellum (T2-mapping-cerebellum-piex-mean) and a specific value in the internal capsule (T2-mapping-internal capsule-X). Additionally, the T2 sequence revealed the corpus callosum-Class1-Signature as a predictive factor (Supplementary Table S1). No significant factors were found in the DTI and T1 mapping sequences for treatment prediction. However, the DKI sequence identified two factors: DKI-cerebellum-Volume and DKI-visual and auditory cortex-Image STD. The T2 mapping sequence indicated two factors: T2-mapping-visual and auditory cortex-Y and T2-mapping-motor and somatosensory cortex-X. Furthermore, the T2 sequence identified corpus callosum-Class0-Signature and internal capsule-Class1-Signature (Supplementary Table S1).

#### 3.8.2 Radiomics scoring (multimodal models)

In the multimodal modeling prediction, T1-mapping-cerebellum-mean was an independent predictive factor. However, additional radiomic features also showed predictive relevance (Supplementary Table S2). For treatment prediction, DKI-visual and auditory cortex-Image STD, along with T2-mapping-visual and auditory cortex-Y, were recognized as independent predictive factors in the multimodal model (Supplementary Table S3).

#### 3.8.3 Comparison of diagnostic performance of unimodal and multimodal models

**Figure 6:**
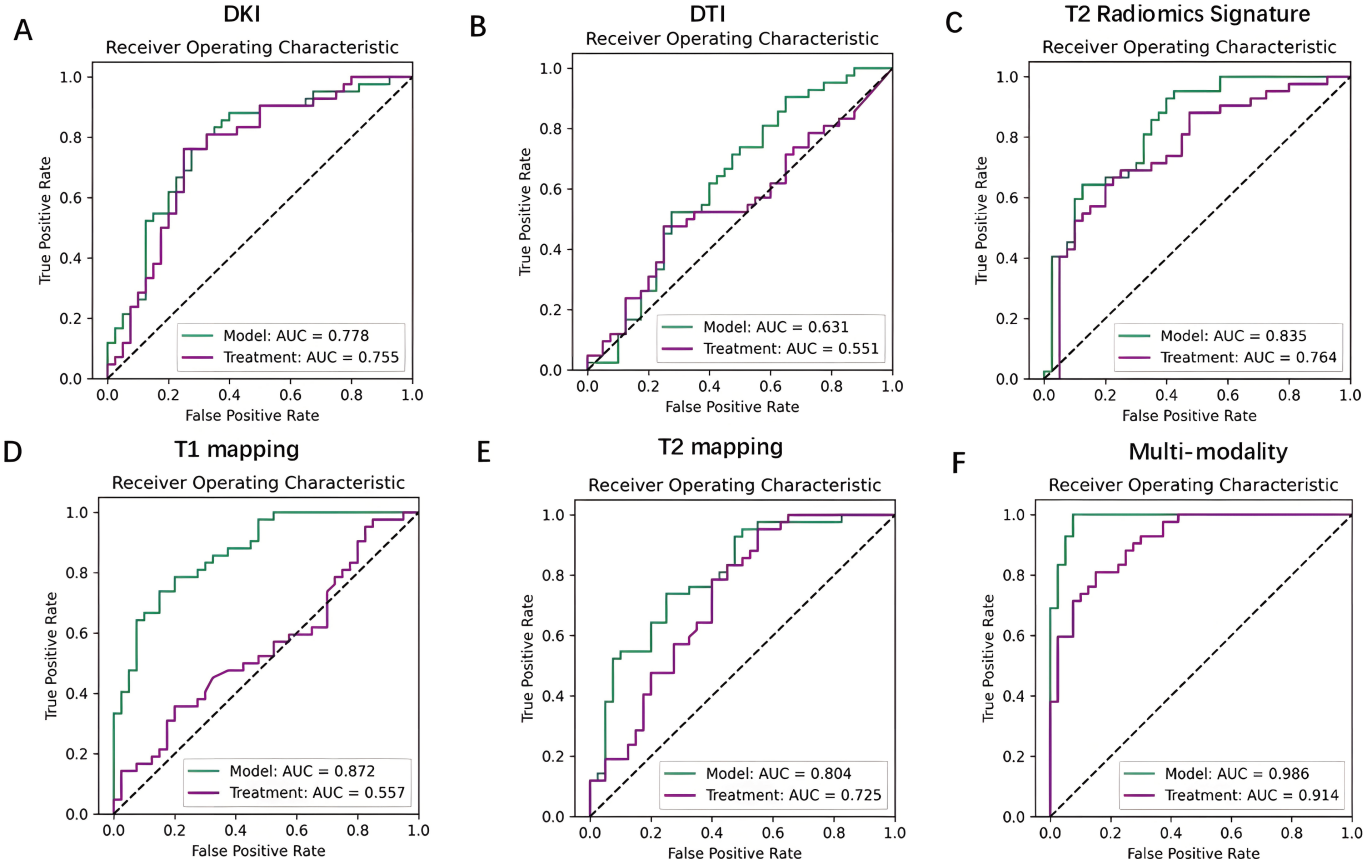
The ROC curves for single-modality and multimodal imaging scores are presented. **(A)** ROC curves for the single-modality DKI. **(B)** ROC curves for the single-modality DTI. **(C)** ROC curves for the single-modality T2 Radiomics Signature. **(D)** ROC curves for the single-modality T1 mapping. **(E)** ROC curves for the single-modality T2 mapping. **(F)** ROC curves for the multimodal imaging scores.

Diagnostic performance comparison between unimodal and multimodal models was conducted through ROC curve analysis, utilizing predictive factors and models from both strategies. The multimodal radiomics scores exhibited high predictive capability for both modeling (AUC = 0.986) and treatment (AUC = 0.914), with the predictive accuracy for modeling surpassing that for treatment. The multimodal model demonstrated superior performance over unimodal models, a finding corroborated by AUC comparison using the DeLong test (Figure 6).

For modeling prediction, the multimodal model showed significantly higher AUC values compared to the unimodal models, including DKI, DTI, T1M, T2M, and T2 sequences (n = 20 rats/group, DeLong test comparing the multimodal model with unimodal models; multimodal model verse DKI model: *Z* = 4.151, *p* < 0.001; multimodal model verse DTI model: *Z* = 5.461, *p* < 0.001; multimodal model verse T1M model: *Z* = 3.332, *p* < 0.001; multimodal model verse T2M model: *Z* = 3.887, *p* < 0.001; multimodal model verse T2 model: *Z* = 3.531, *p* < 0.001). Similarly, for treatment prediction, the multimodal model demonstrated significantly higher AUC values compared to the unimodal models (n = 20 rats/group, DeLong test comparing the multimodal model with unimodal models; multimodal model verse DKI model: *Z* = 3.016, *p* = 0.003; multimodal model verse DTI model: *Z* = 4.813, *p* < 0.001; multimodal model verse T1M model: *Z* = 5.225, *p* < 0.001; multimodal model verse T2M model: *Z* = 3.547, *p* < 0.001; multimodal model verse T2 model: *Z* = 3.287, *p* = 0.001).

**Figure 7:**
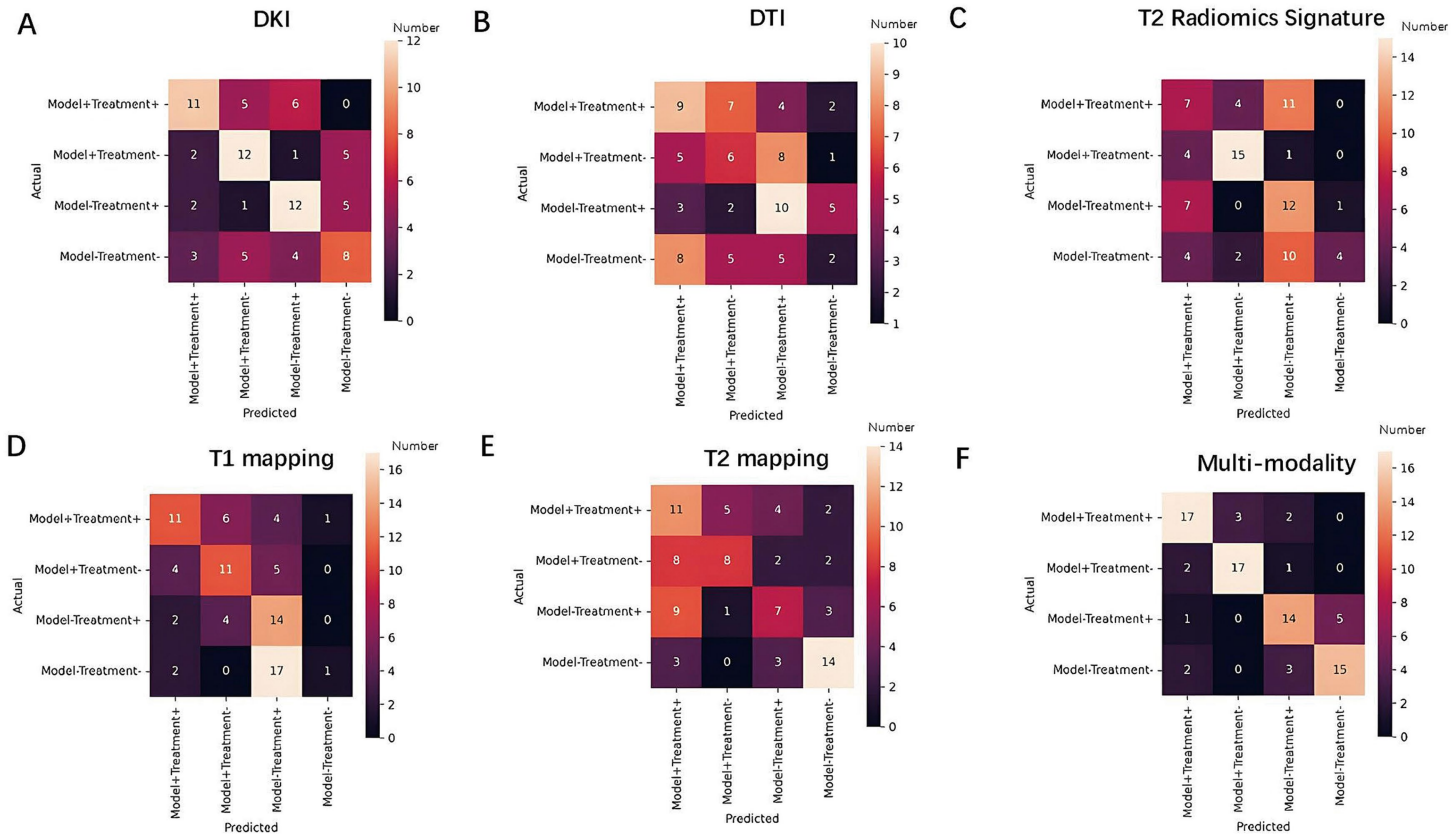
Matrix plot of single-modality and multimodal models: This plot shows the overall classification performance and characteristics of each model based on the color distribution. **(A)** Matrix plot of single-modality in DKI. **(B)** Matrix plot of single-modality in DTI. **(C)** Matrix plot of single-modality in T2 Radiomics Signature. **(D)** Matrix plot of single-modality in T1 mapping. **(E)** Matrix plot of single-modality in T2 mapping. **(F)** Matrix plot of single-modality in multimodal model.

**Figure 8:**
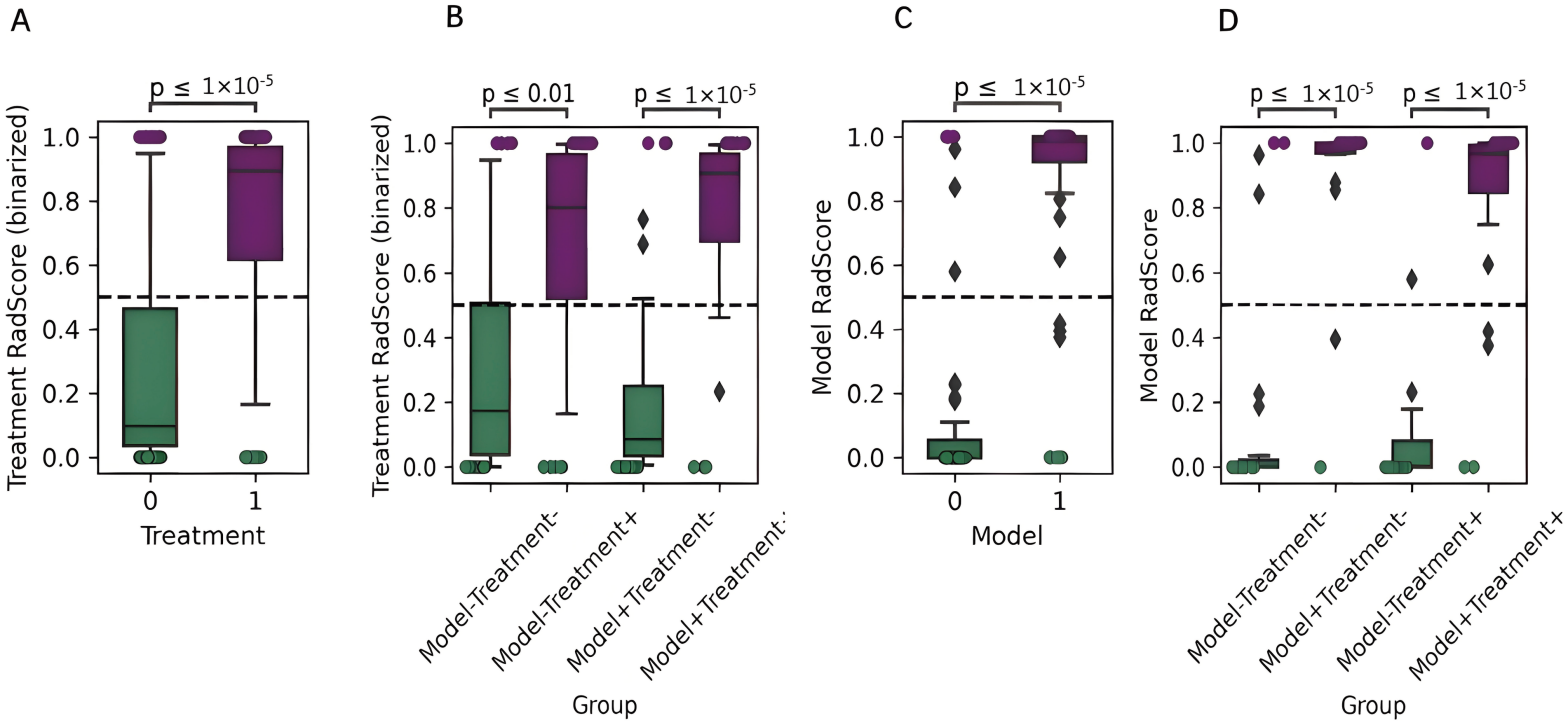
Comparison of two imaging biomarkers in the multimodal model (modeling and treatment biomarkers): **(A)** Comparison of treatment biomarker scores between treatment subgroups in the multimodal model, where 0 represents no treatment, and 1 represents treatment. **(B)** Comparison of treatment biomarker scores for all tested rats in the multimodal model. **(C)** Comparison of treatment biomarker scores between the modeling subgroups in the multimodal model, where 0 represents no treatment and 1 represents treatment. **(D)** Comparison of modeling biomarker scores for all tested rats in the multimodal model.

The multimodal radiomics scores demonstrated superior accuracy, sensitivity, and specificity in binary classification performance compared to the unimodal models, specifically in modeling (accuracy: 0.927; sensitivity: 0.929; specificity: 0.925) and treatment predictions (accuracy: 0.805; sensitivity: 0.810; specificity: 0.800) (Figure 7; Supplementary Table S4). The multimodal model also provided more accurate predictions across the entire sample and various subgroups, outperforming unimodal models (Figure 8; Supplementary Figures S11–S15).

#### 3.8.4 Correlation of multimodal radiomics scores with behavioral and developmental features

Multimodal radiomics scores were associated with specific behavioral features under different target conditions (Supplementary Table S5). Regarding developmental indices, significant associations were only observed with modeling radiomics scores (Supplementary Table S6). Correlation analysis between developmental features and selected measurement and radiomic features from the DKI and DTI sequences revealed strong associations with statistically significant differences, alongside notable correlations for other sequences (Supplementary Figures S16a–e).

## 4 Discussion

Children with ASD often experience developmental delays (Khera et al., 2022) and a concurrent decline in selenium levels (Mehta et al., 2021), suggesting a potential link between selenium deficiency and structural brain abnormalities in ASD. Hypoplasia of the corpus callosum (Wu et al., 2018) and microstructural changes in the internal capsule (Phiel and Klein, 2001; Rutishauser et al., 2015) are commonly observed in ASD, showing the need to investigate selenium’s role in redox balance maintenance and its potential impact on the pathogenesis of ASD due to oxidative stress.

Employing an animal model, our research conducted developmental and behavioral assessments alongside multi-sequence imaging models, revealing that selenium supplementation ameliorates developmental delays and behavioral abnormalities. Using radiomic analytical methods, we identified independent diagnostic factors for evaluating the therapeutic effects of ASD modeling and selenium treatment. Additionally, we observed that selenium supplementation could ameliorate certain abnormal brain structures, further supporting the notion that selenium exerts a beneficial impact on brain structure and function in ASD.

Observations from our study showed that VPA-treated rat pups exhibited developmental delays similar to those seen in human cases, including in body weight, tail length, and motor and balance functions. These findings reveal the teratogenic and neurotoxic effects of prenatal VPA exposure, which may contribute to developmental delays and is known to affect neural tube development (Fathe et al., 2014).

The study also replicated developmental abnormalities associated with epigenetic changes induced by environmental factors linked to autism (Hernández-Díaz et al., 2024). In addition to weight and tail length, delayed eye-opening and geotaxis functions were observed, which were consistent with previous findings (Pal et al., 2022). These characteristics are shared between human populations and animal models.

The results from the open-field experiment indicated that rats in the ASD group spent less time in the central area and exhibited increased urination and defecation, signifying the presence of anxiety within the ASD animal model (Williams et al., 2005). These findings support the hypothesis that autism-like behaviors, such as anxiety, are associated with prenatal exposure to VPA in rats (Guerrin et al., 2022). Post selenium supplementation, the rats displayed an increased duration spent in the central area and a reduction in spontaneous behaviors, suggesting that selenium can mitigate anxiety-like symptoms in ASD rats. Similarly, the marble burying test corroborated the open-field test results, further validating that selenium can ameliorate anxiety-related behaviors in ASD rats.

In the three-chamber social test, rats across all groups demonstrated significant social behaviors during the social interaction and preference phases, with the control group exhibiting stronger social engagement (Hinkley et al., 2012), aligning with prior studies. This behavior shows the mediating role of the amygdala and the prefrontal-temporal cortex in social interactions, which maintain functional connections with the basal ganglia, hippocampus, and other regions, thus facilitating social cognition and driving social (Hollocks et al., 2019) behaviors. However, no significant differences were noted in the social or social preference index post-selenium intervention, contrasting with earlier research. This variance might be attributed to the relatively short duration of the selenium treatment, potentially insufficient for effectively activating the relevant brain regions.

Rats in the ASD group demonstrated impaired learning and memory function in the novel object recognition test, an effect that selenium supplementation improved. This observation aligns with the memory deficits frequently noted in children with ASD (AlAbdi et al., 2023). This outcome further shows the critical roles of the hippocampus and prefrontal cortex in learning and memory, suggesting that the communication and memory deficits characteristic of autism are primarily linked with these areas (Banker et al., 2021).

Based on the observed behavioral outcomes, selenium supplementation effectively improves stereotypic behavior, anxiety, and learning and memory performance in rats. This suggests a beneficial impact of selenium on the cognitive functions of the corpus callosum (Bezgin et al., 2024) and on the emotional processing and memory functions of the hippocampus and amygdala (Wang et al., 2014). Current studies propose that selenium may protect these brain regions by enhancing antioxidant enzyme activity, mitigating oxidative stress-induced damage, and reducing inflammation (Adiguzel et al., 2024). However, its limited effectiveness on social behavior may stem from the inherent complexity of social interactions. Social behavior is influenced by the hippocampus, frontal lobe, and other regions, such as the cerebellum and dorsal raphe nucleus, integral to neural networks that project to the frontal lobe and other related areas (Carta et al., 2019; Emens et al., 2021). If selenium intervention does not fully restore the structure and function of these areas or if the interconnections between them remain compromised, improvements in social behavior may be constrained.

To further explore the impact of selenium on behavioral improvements, future research will utilize neuroimaging techniques to analyze the relationship between selenium and the pathogenesis of ASD, focusing on its mechanisms and effects on relevant brain regions.

Using multimodal MRI data, we developed both unimodal and multimodal radiomics models, which have shown robust diagnostic and assessment performance. A strong correlation exists between multimodal models and specific behavioral and developmental indicators, thus highlighting the role of different brain regions associated with various predictive outcomes. Notably, after selenium treatment, structural and functional improvements were observed in specific brain regions, providing new insights for the treatment of children with ASD.

Notably, the multimodal predictive model identified the cerebellum as an independent diagnostic factor for ASD modeling. The findings suggest that cerebellar changes could be a sensitive indicator for the diagnosis of autism. Previous research has shown a reduction in cerebellar volume and Purkinje cell count in ASD, which may lead to decreased GABA-related antibody activity in Purkinje fibers, promoting increased spontaneous activity and reduced social behavior—key symptoms of autism (Fujita et al., 2024). This also reveals a significant link between abnormalities in cerebellar structure and function and the development of autism. Furthermore, the cerebellum’s extensive neural fibers project to the cortex and limbic system, which regulate emotional and cognitive functions (Anderson et al., 2011). When environmental factors or ASD susceptibility genes affect the structural integrity of the cerebellum and its associated neural pathways, the resulting disruption may impair higher cognitive and social functions (Deemyad et al., 2022).

Our study identified the cerebellum as a critical predictive factor for assessing the impact of selenium treatment on brain structures in ASD. Previous research has demonstrated a correlation between cerebellar size and selenium levels in newborns, suggesting the cerebellum’s potential as a biomarker and target “organ” for monitoring changes in trace elements such as selenium (Močenić et al., 2019). These findings further highlight the potential therapeutic role of selenium supplementation in treating ASD.

The visual–auditory cortex emerged as a crucial region among the independent diagnostic factors associated with selenium treatment in our multimodal model. Earlier studies have indicated that individuals with autism may experience selective atrophy in the temporal lobe and delayed neuronal activity (Čobanová et al., 2017). Additionally, reduced activity in the insular cortex of children with ASD has been linked to poorer auditory phrase recognition performance (Cechella et al., 2014). Given that the visual–auditory cortex primarily governs auditory perception and speech processing, abnormalities in these regions may lead to deficits in auditory and speech processing and reduced speech sensitivity, exacerbating social impairments in individuals with autism (Anwer and Hassanin, 2023). These findings highlight the pivotal role of the visual–auditory cortex in the pathophysiology of ASD.

Animal studies have shown a correlation between selenium levels and the protection of cortical structures and microcircuits from damage, emphasizing selenium’s protective role against such injuries (Roseni Mundstock Dias et al., 2014). A study using an animal model exposed to heavy metal ions demonstrated that selenium could mitigate neuronal damage and apoptosis by reducing oxidative stress induced by environmental factors (Močenić et al., 2019). In the visual cortex (layer V), selenium has been shown to protect pyramidal neurons by alleviating mitochondrial and DNA dysfunction (Clausi et al., 2021). Furthermore, selenium reduces lipid peroxidation and oxidative stress in the visual and auditory cortices, preserving the structural and functional integrity of pyramidal and granule cells. It also promotes the production of antioxidant enzymes, thereby ensuring genomic stability (Silvestrini et al., 2020).

Given the observed protective effects of selenium in ASD animal models and the physiological functions of the visual–auditory cortex, selenium supplementation has been found to significantly enhance the structure and function of the visual–auditory cortex in ASD, leading to notable behavioral improvements. The brain processes sensory stimuli from vision and hearing more clearly and prioritizes these over stimuli from other regions (Nanay, 2018). As a result, improvements in visual and auditory functions are more readily observable and quantifiable. Furthermore, a synergistic interaction among the visual, auditory, and sensorimotor regions has been demonstrated by both structural and functional studies (Huang et al., 2015; Comstock et al., 2021; Si et al., 2022). For example, when one sensory modality is impaired, the brain compensates by enhancing other sensory modalities, ensuring the coordination and integrity of motor perception, spatial orientation, and motor regulation. Additionally, research on brain networks has indicated that the visual–auditory cortex is involved in frontoparietal network tasks related to memory, attention, visual processing, cognitive control, proprioception, and pain management (Costumero et al., 2018). Given the extensive role of the visual–auditory cortex in brain structure and function, its marked structural and functional improvements following treatment offer a strong rationale for identifying related metrics of this region as independent diagnostic factors for selenium treatment in ASD within our multimodal model.

Notably, the unimodal model identified regions such as the corpus callosum, internal capsule, and amygdala as key areas for predictive modeling and selenium treatment. However, the hippocampus only exhibited predictive relevance for ASD diagnosis and did not show responsiveness to selenium treatment. Prior studies have indicated that VPA exposure leads to reduced thickness in the amygdala and hippocampal CA1 region of rats (Rønne et al., 2017), as well as decreased dendritic complexity in Purkinje cells (Schnider et al., 2013). Children with ASD also exhibit reduced hippocampal volume, decreased cell density and neuronal count in the CA1 region, diminished dendritic branching (Müller, 2011; Isaev et al., 2024), and disrupted neuronal maturation (McLaughlin et al., 2018). These findings suggest that the hippocampus is particularly vulnerable to changes associated with ASD, which is consistent with our observations. As a crucial brain region for spatial memory and emotional regulation, the hippocampus contains relatively high selenium levels, making it sensitive to fluctuations in brain selenium concentrations (Marini et al., 2023). However, our study did not confirm this sensitivity, and although the hippocampus did not emerge as a significant factor in our treatment prediction, its potential role should not be overlooked. This lack of significance may be attributed to factors such as the choice of animal models, imaging sequence, and scan timing, which could influence the results. Additionally, this might explain why no significant differences were observed between the selenium intervention group and the ASD model group during the social phase of the three-chamber social test. Selenium, an inexpensive and easily accessible dietary supplement found in many commonly consumed foods, holds promise as a straightforward and practical therapeutic approach in clinical research. This study also aims to provide a theoretical foundation for future investigations into the role of selenium in the oxidative stress mechanisms underlying ASD.

### 4.1 Limitation

There were several limitations in our study. First, the multimodal imaging sequence did not include functional MRI scans, which prevented us from assessing the effects of selenium on brain regions under both resting and task-related conditions. Second, although the unimodal model identified several brain regions with diagnostic significance for ASD modeling and selenium treatment, such as the hippocampus, corpus callosum, internal capsule, and amygdala, these regions did not emerge as strong predictors in the multimodal model. This discrepancy may be attributed to the limited sample size, which necessitates further exploration of potential molecular pathways, constituting the third limitation of our study. Nonetheless, these findings do not diminish the importance or potential research value of these brain regions in ASD modeling and selenium treatment.

Our study demonstrates that multimodal imaging models offer significantly superior diagnostic and treatment evaluation capabilities compared to unimodal models. These models provide a comprehensive qualitative and quantitative assessment of brain changes. By combining multimodal imaging with behavioral assessments and developmental history, our research enables further exploration into the relationship between selenium and oxidative stress. Additionally, multimodal omics can guide clinical approaches to selenium therapy. The multimodal imaging model identified the cerebellum as a sensitive region in the ASD model, whereas the multimodal omics model highlighted the visual–auditory cortex as a key area influenced by selenium treatment. These insights enhance our ability to predict the effects of selenium on brain structure and function. Furthermore, these findings establish a potential observational region in the brain for the diagnosis and intervention of ASD in humans, contributing novel perspectives to the field of ASD imaging research.

## Data Availability

The raw data supporting the conclusions of this article will be made available by the authors, without undue reservation.

## Supplementary material

Supplementary Data Sheet Screening and performance evaluation results of multimodal imaging features in different brain regions.
