# Supplementary material for: Multimodal MRI-based radiomics in an ASD rat model: investigating brain structural changes and the neuroprotective effects of selenium
Source: Front Neurosci. 2025 Oct 8;19:1651220. doi: 10.3389/fnins.2025.1651220 (PMC12540317; doi:10.3389/fnins.2025.1651220)
Supplement: Supplementary file 1 [file Data_Sheet_1.DOCX]

**Supplementary materials**

^*^Clustering analysis is performed only when multiple related features are present within a region, followed by a correlation matrix plot after clustering. The hierarchical clustering method, specifically the agglomerative hierarchical clustering approach, was employed. This bottom-up technique constructs a dendrogram to illustrate the layer-by-layer relationships within the data clusters. ^#^Prediction targets are modeling and treatment goals, comparing differences between the model and control groups, and between treated and untreated groups. (a) Comparison of treatment biomarker scores in the treatment subgroups using the single-sequence model, where 0 represents no treatment and 1 represents treatment. (b) Comparison of treatment biomarker scores for all tested rats in the single-sequence model. (c) Comparison of treatment biomarker scores in the modeling subgroups using the single-sequence model, where 0 represents no treatment and 1 represents treatment. (d) Comparison of modeling biomarker scores for all tested rats in the single-sequence model.


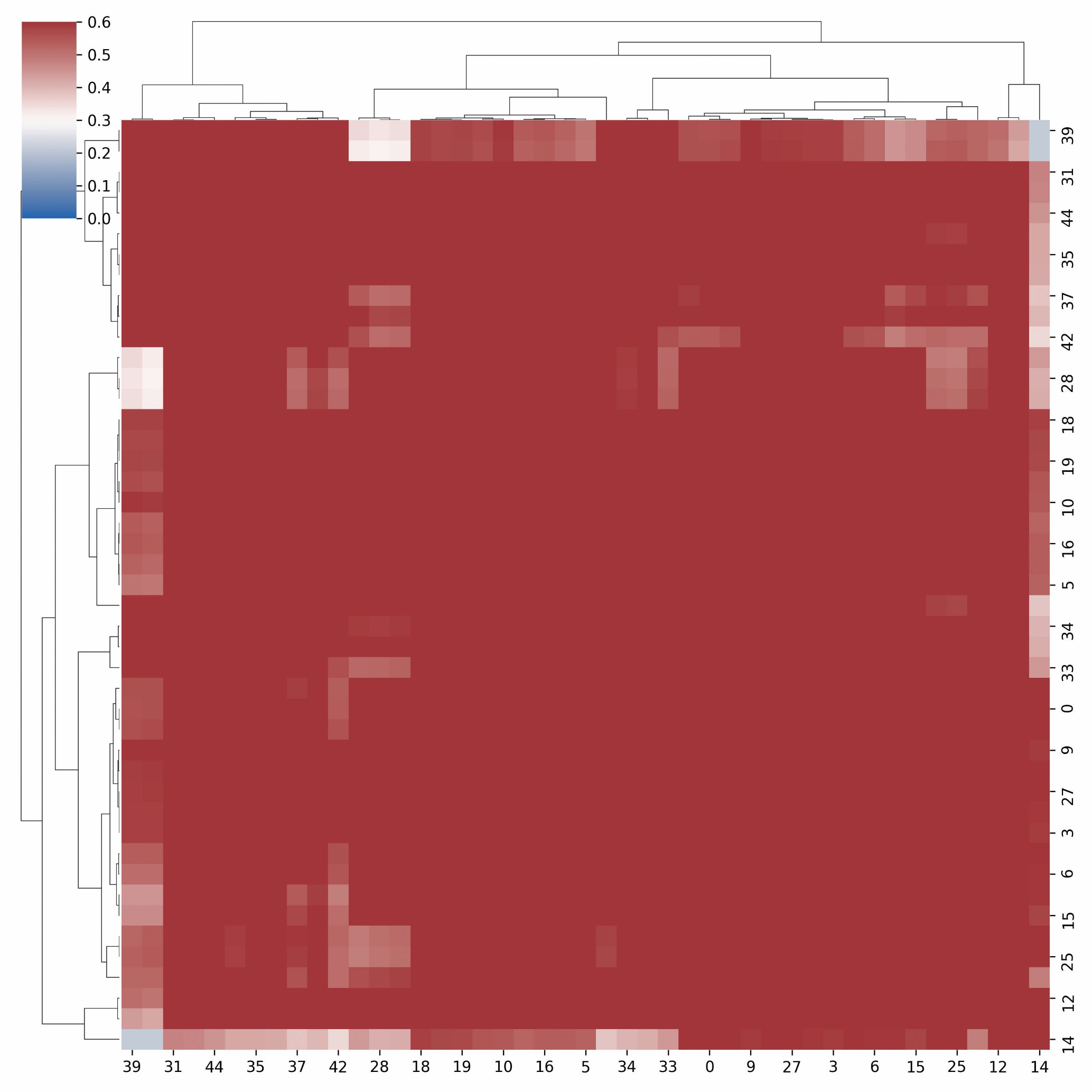


**Figure S1** Correlation matrix of imaging features in the sensory-motor cortex region.

**
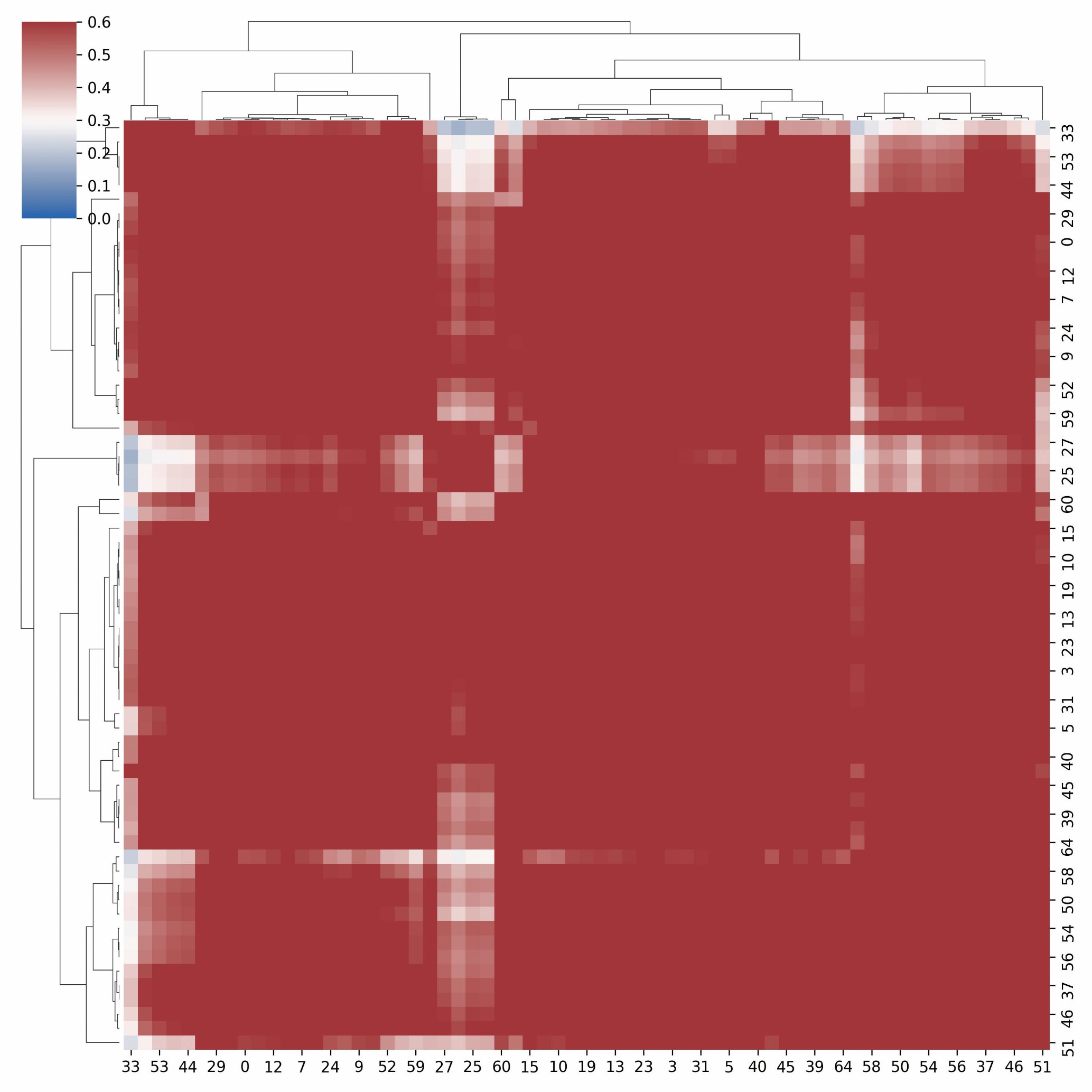
**

**Figure S2** Correlation matrix of imaging features in the cerebellum region.

**
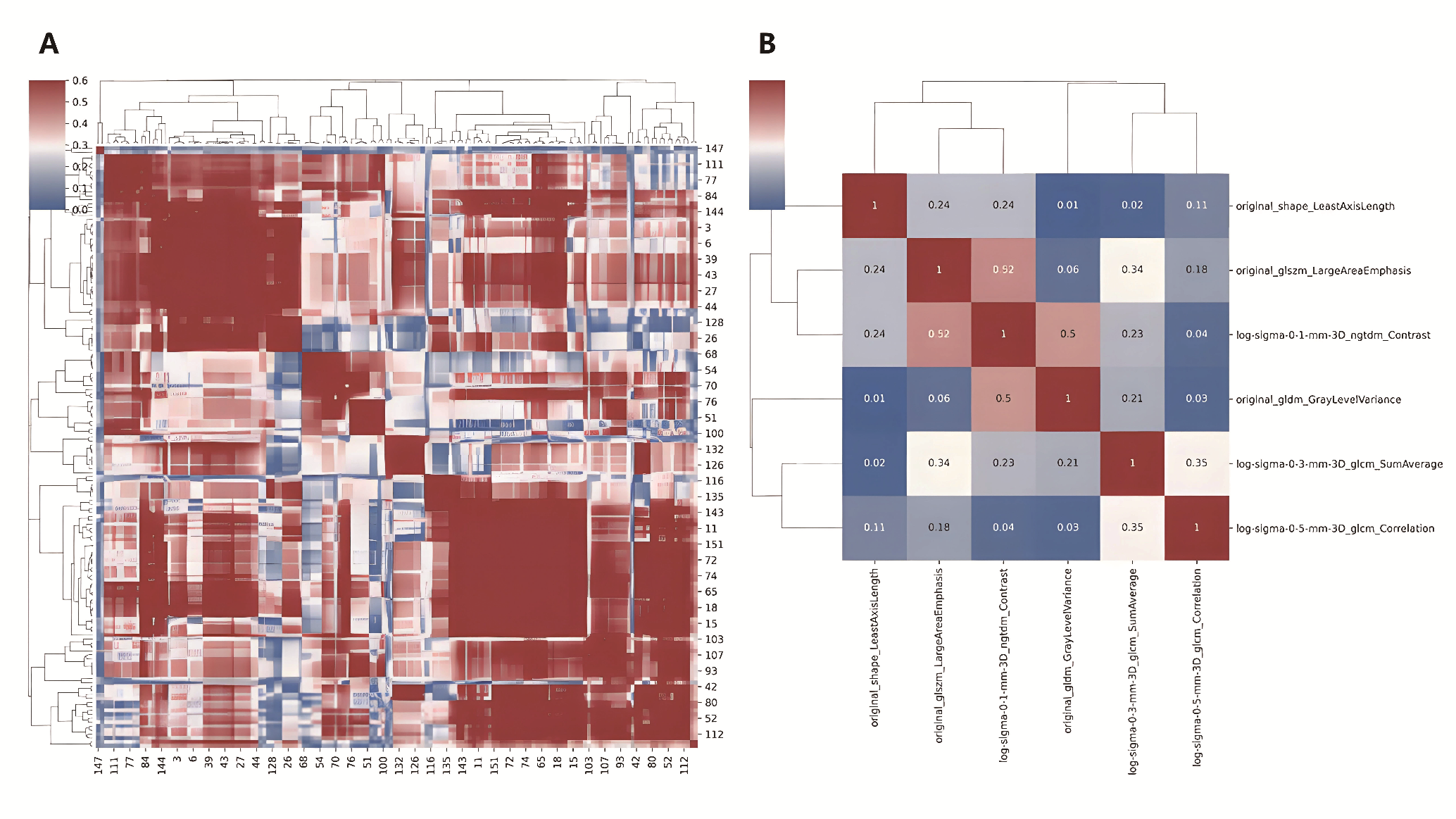
**

**Figure S3** Correlation matrices of imaging features in the corpus callosum region: (a) correlation matrix of imaging features before clustering analysis; (b) correlation matrix of imaging features after clustering analysis.

**
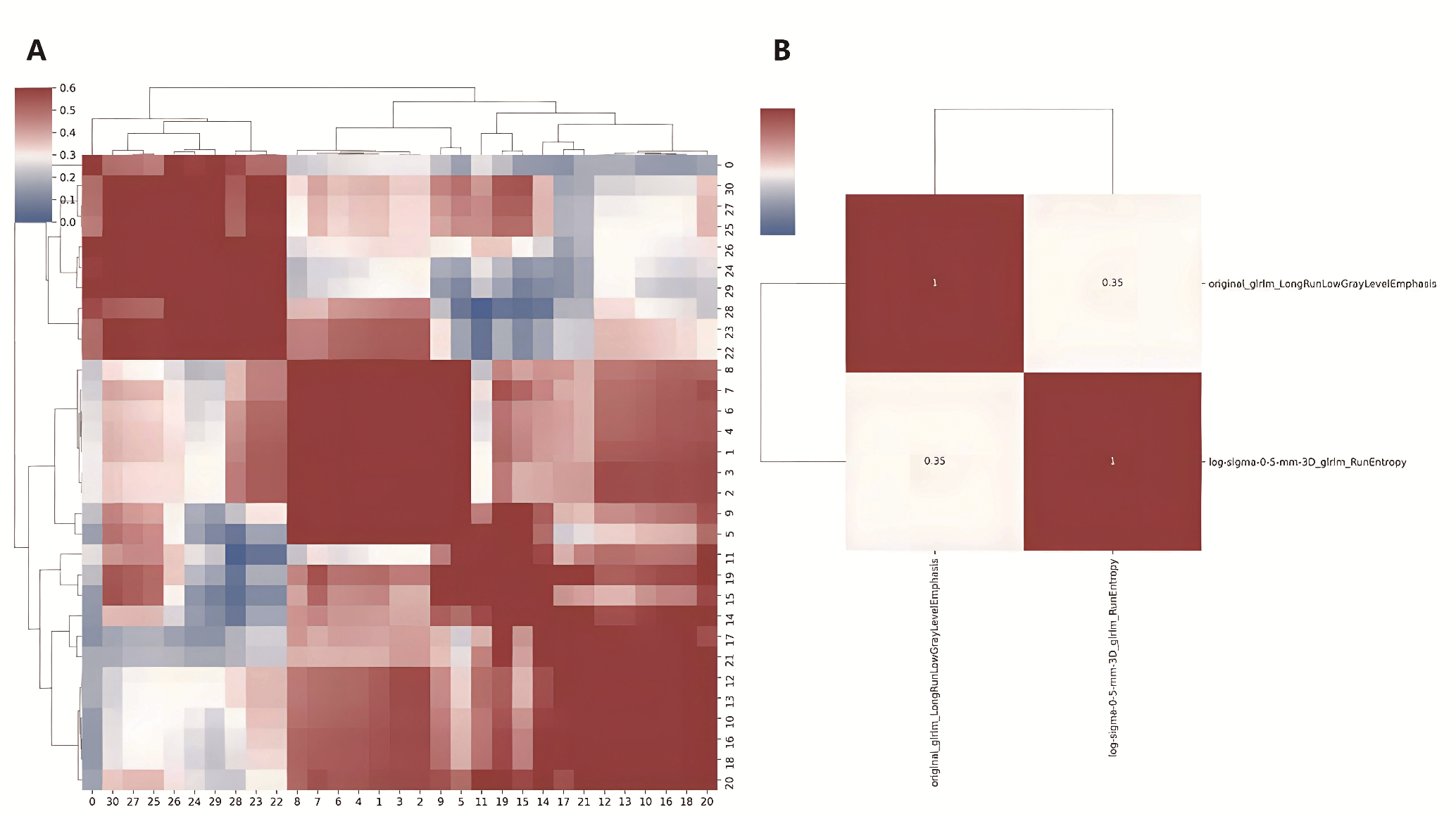
**

**Figure S4** Correlation matrices of imaging features in the internal capsule region: (a) correlation matrix of imaging features before clustering analysis; (b) correlation matrix of imaging features after clustering analysis.

**
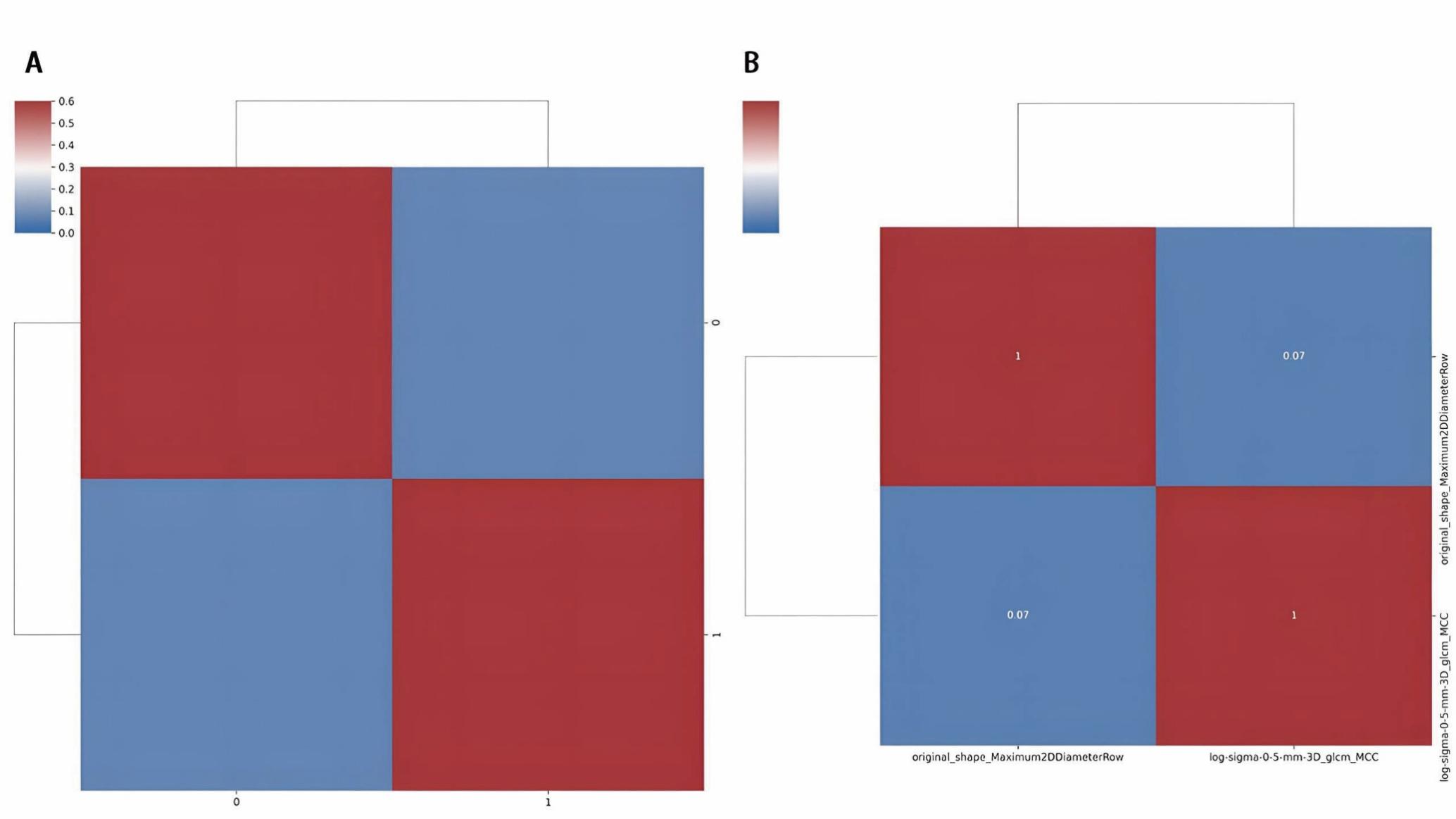
**

**Figure S5** Correlation matrices of imaging features in the hippocampal region: (a) Correlation matrix of imaging features before clustering analysis; (b) Correlation matrix of imaging features after clustering analysis.


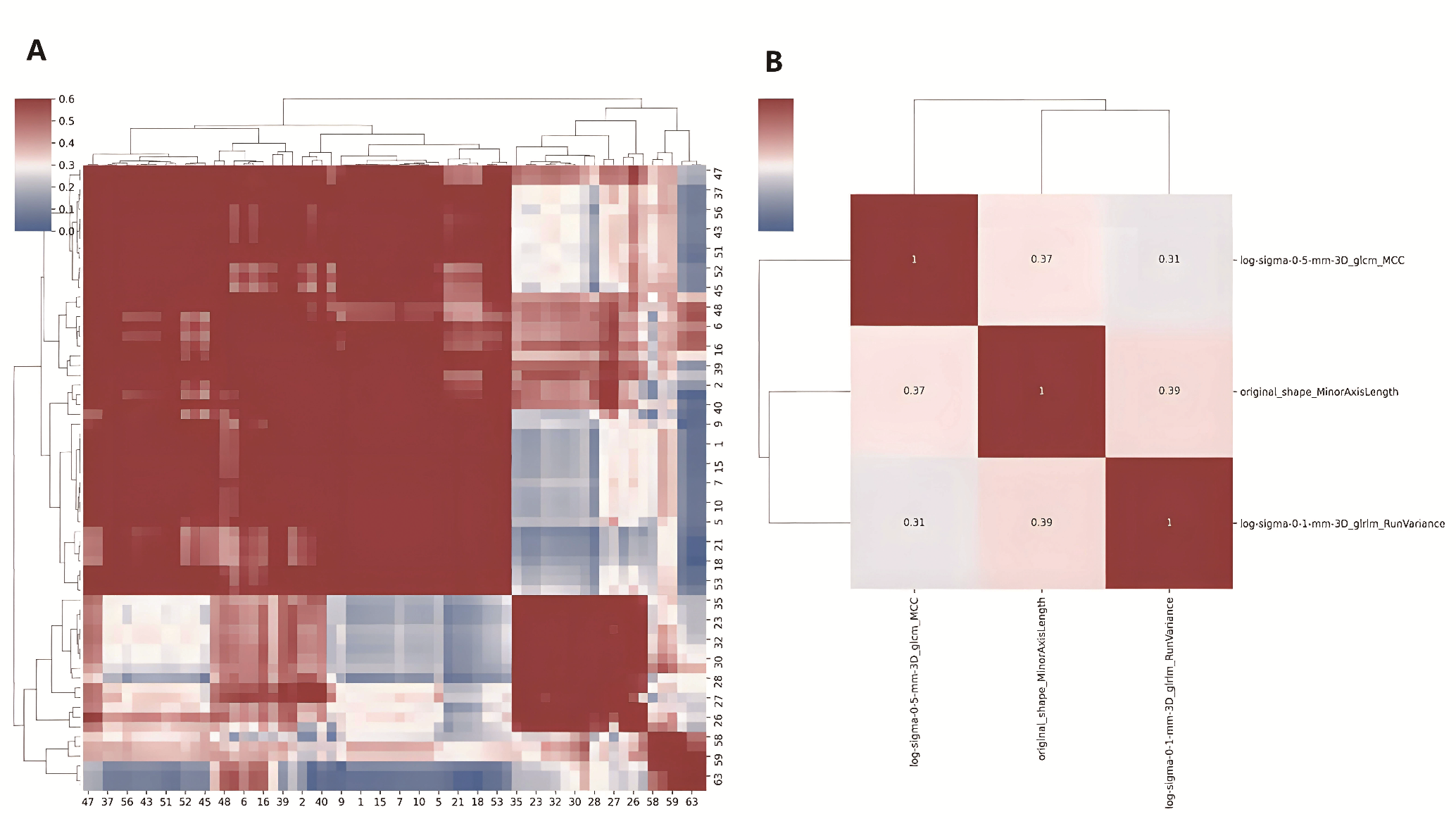


**Figure S6** Correlation matrices of imaging features in the visual-auditory cortex region: (a) correlation matrix of imaging features before clustering analysis; (b) correlation matrix of imaging features after clustering analysis.

**
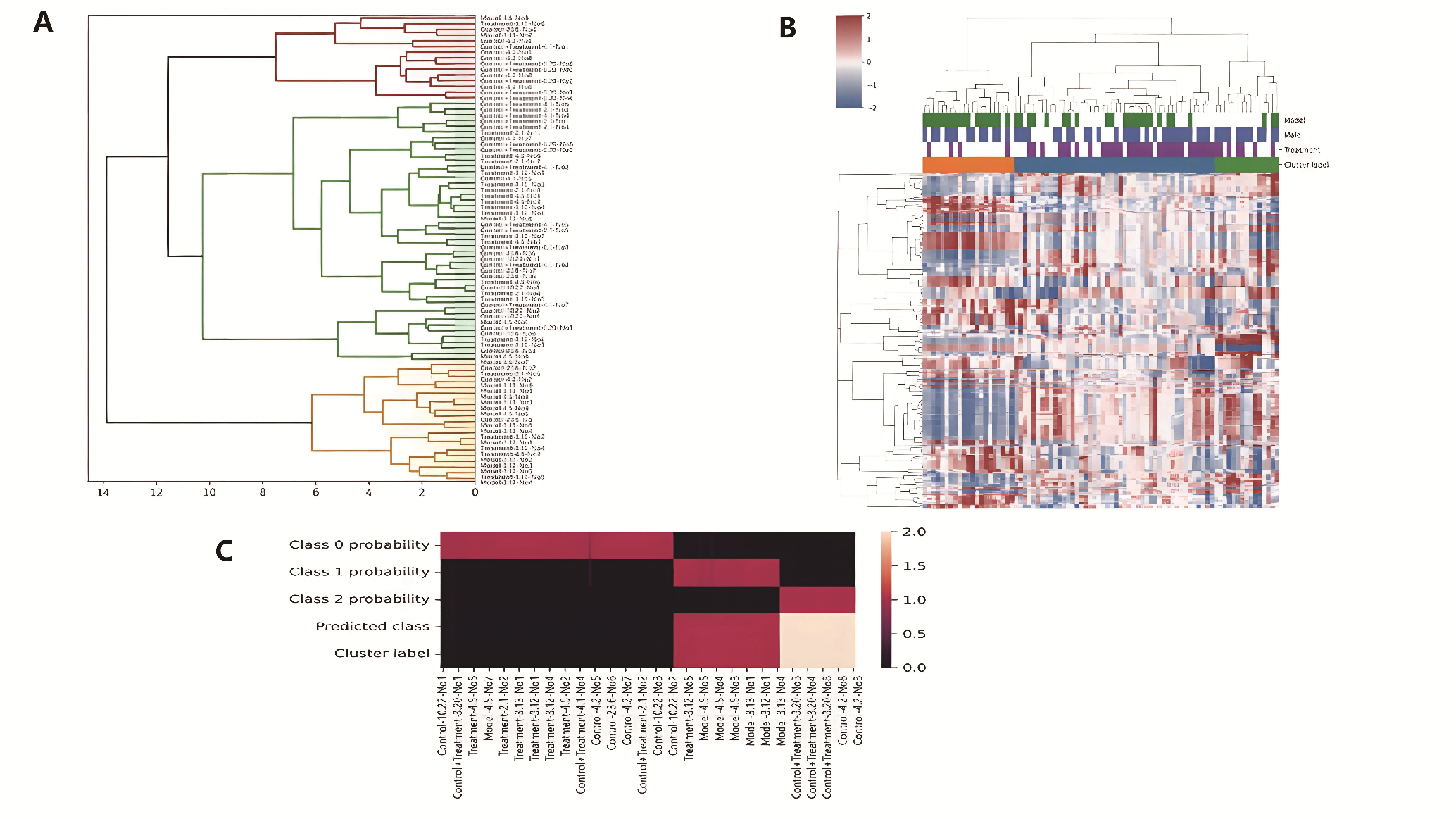
**

**Figure S7** Clustering and predictive analysis in the corpus callosum region: (a) dendrogram of sample clustering; (b) heatmap of imaging features correlated with behavior after clustering analysis; (c) model prediction results of predictive factors in the corpus callosum region.

**
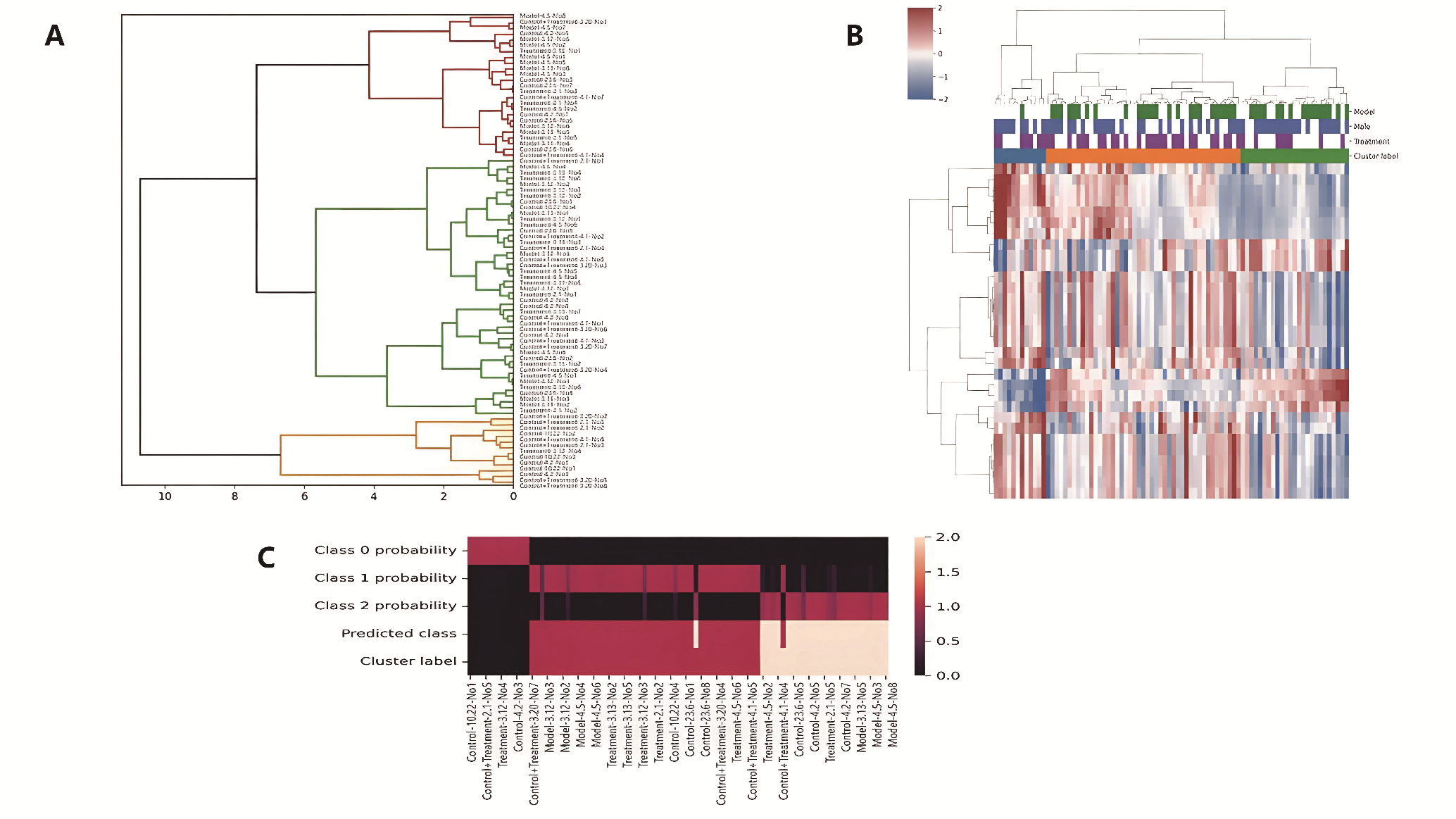
**

**Figure S8** Clustering and predictive analysis in the internal capsule region: (a) dendrogram of sample clustering; (b) heatmap of imaging features correlated with behavior after clustering analysis; (c) model prediction results of predictive factors in the internal capsule region.

**
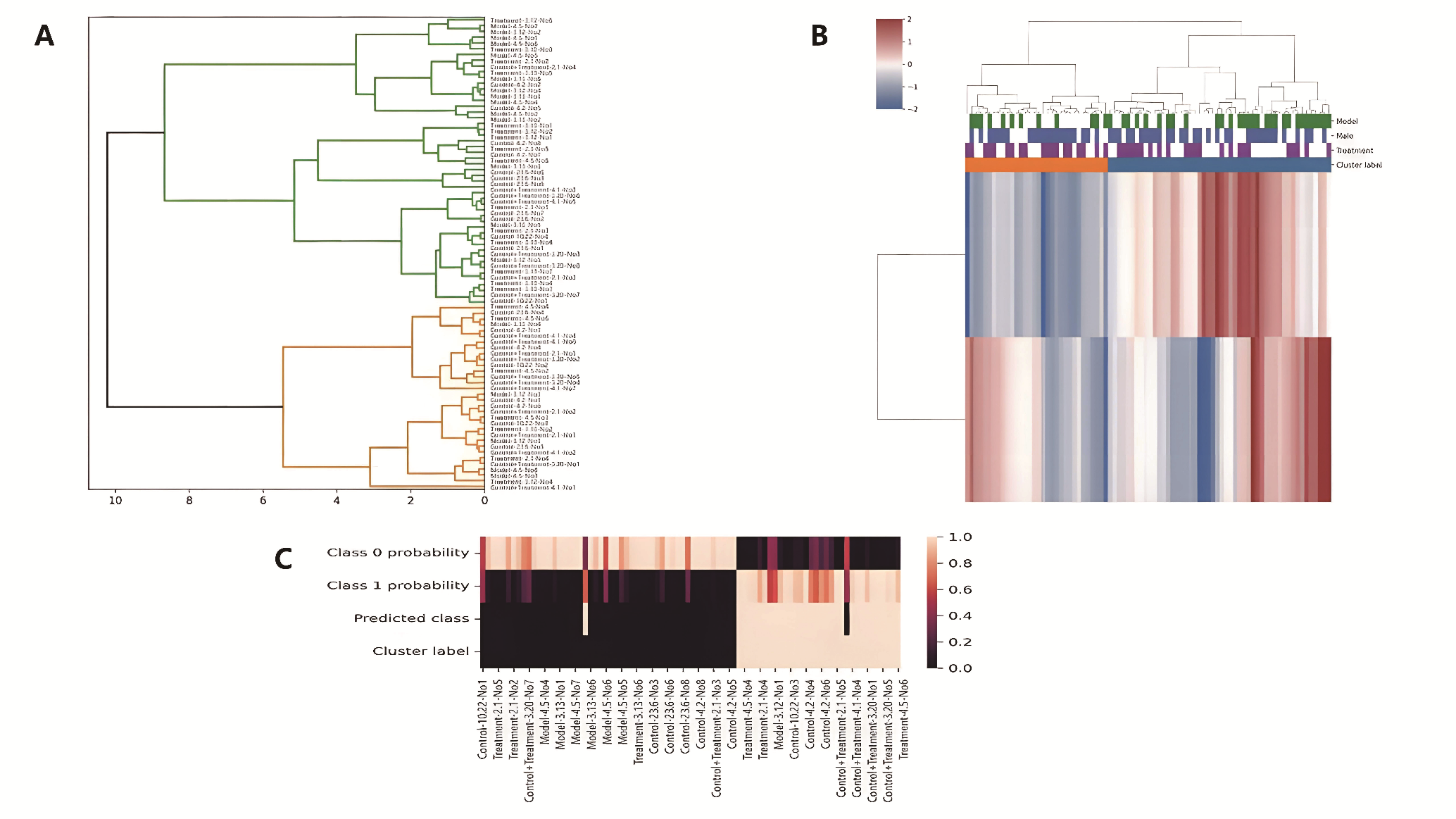
**

**Figure S9** Clustering and predictive analysis in the hippocampus region: (a) dendrogram of sample clustering; (b) heatmap of imaging features correlated with behavior after clustering analysis; (c) model prediction results of predictive factors in the hippocampus region.

**
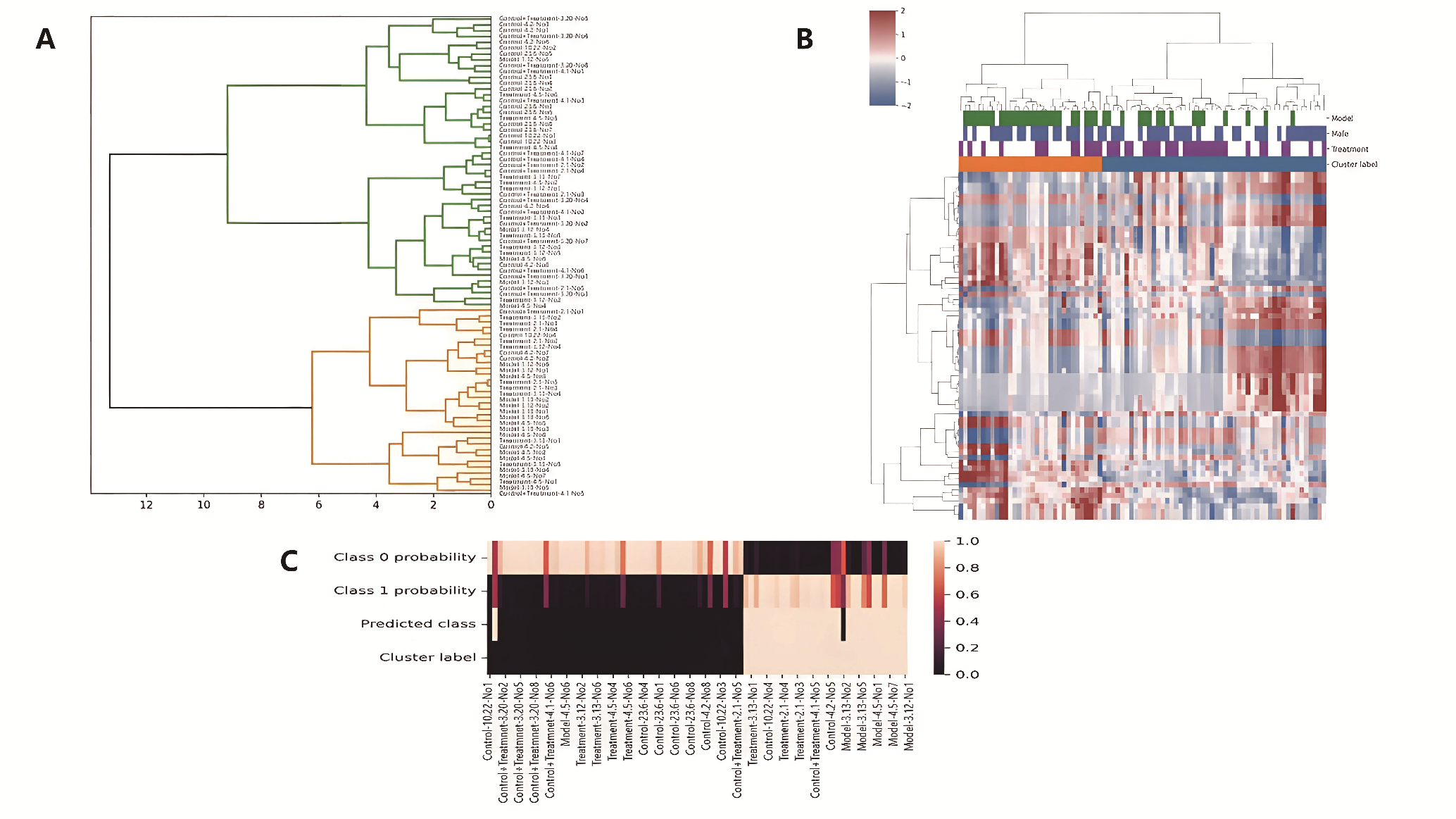
**

**Figure S10** Clustering and predictive analysis in the visual-auditory cortex region: (a) dendrogram of sample clustering; (b) heatmap of imaging features correlated with behavior after clustering analysis; (c) model prediction results of predictive factors in the visual-auditory cortex region.


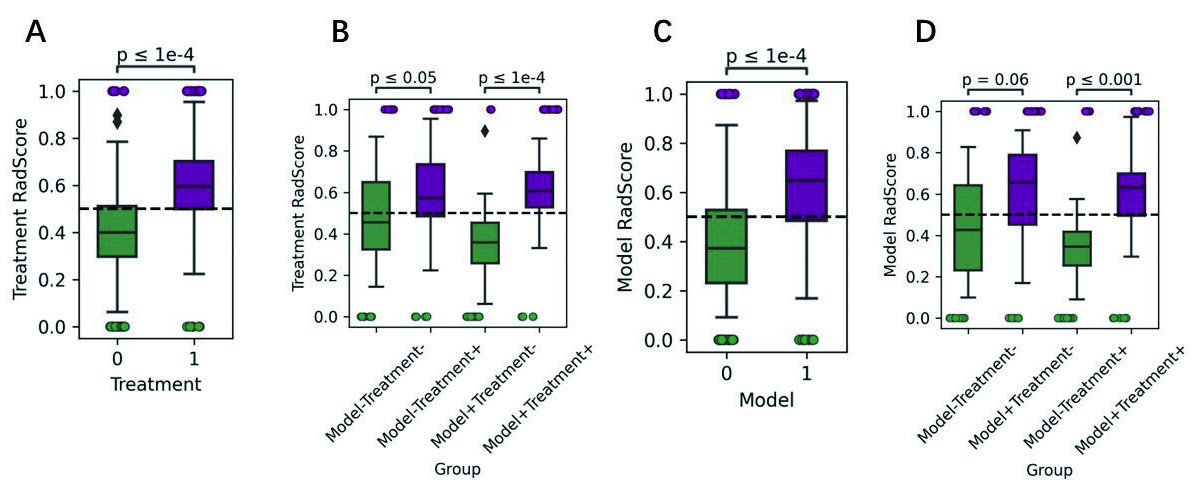


**Figure S11** Comparison of the DKI sequence model in modeling and treatment prediction targets across different subgroups.

**
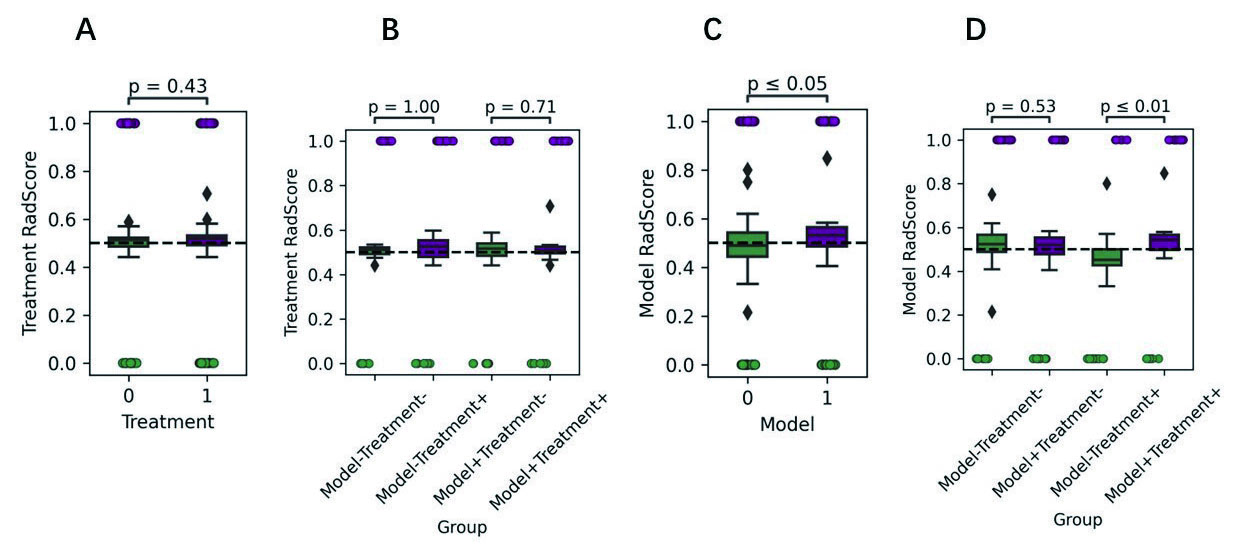
**

**Figure S12** Comparison of the DTI sequence model in modeling and treatment prediction targets across different subgroups.


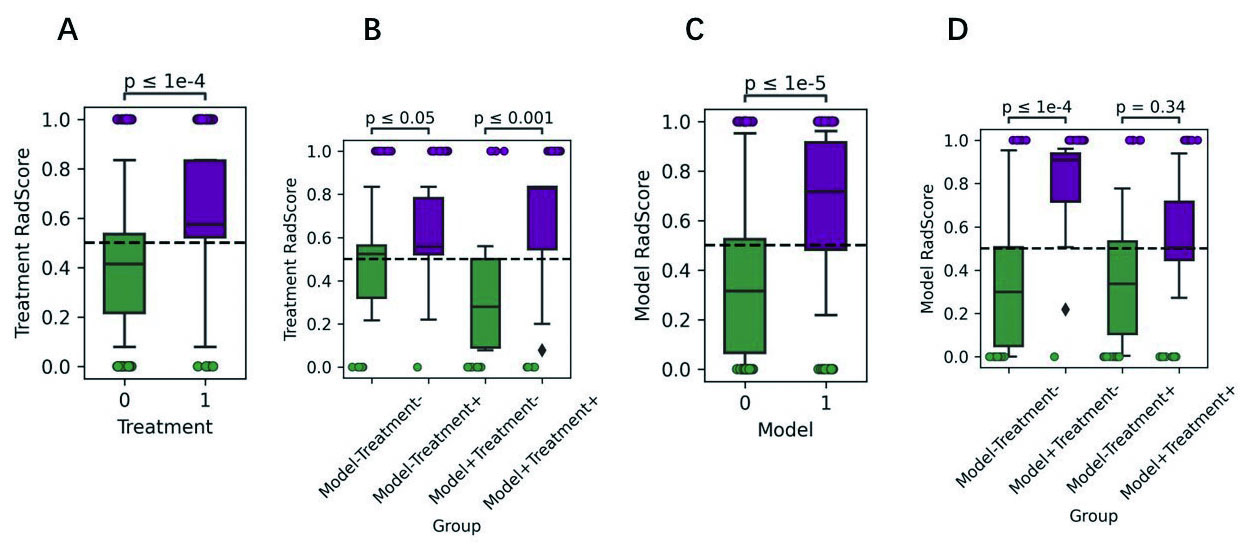


**Figure S13** Comparison of the T2 sequence model in modeling and treatment prediction targets across different subgroups.


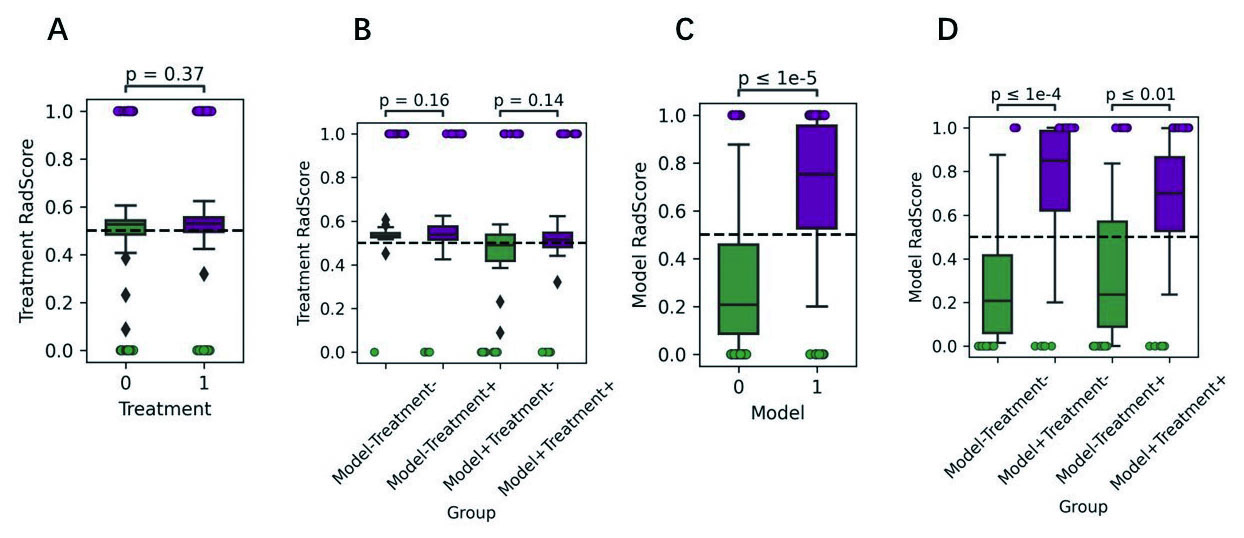


**Figure S14** Comparison of the T1 mapping sequence model in modeling and treatment prediction targets across different subgroups.


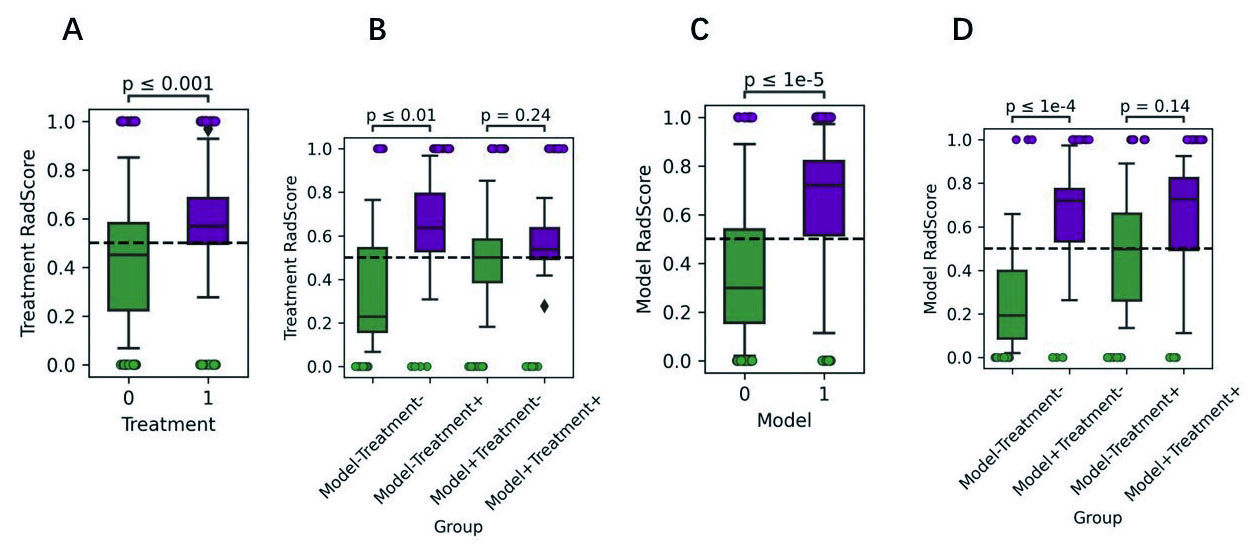


**Figure S15** Comparison of the T2 mapping sequence model in modeling and treatment prediction targets across different subgroups.

**
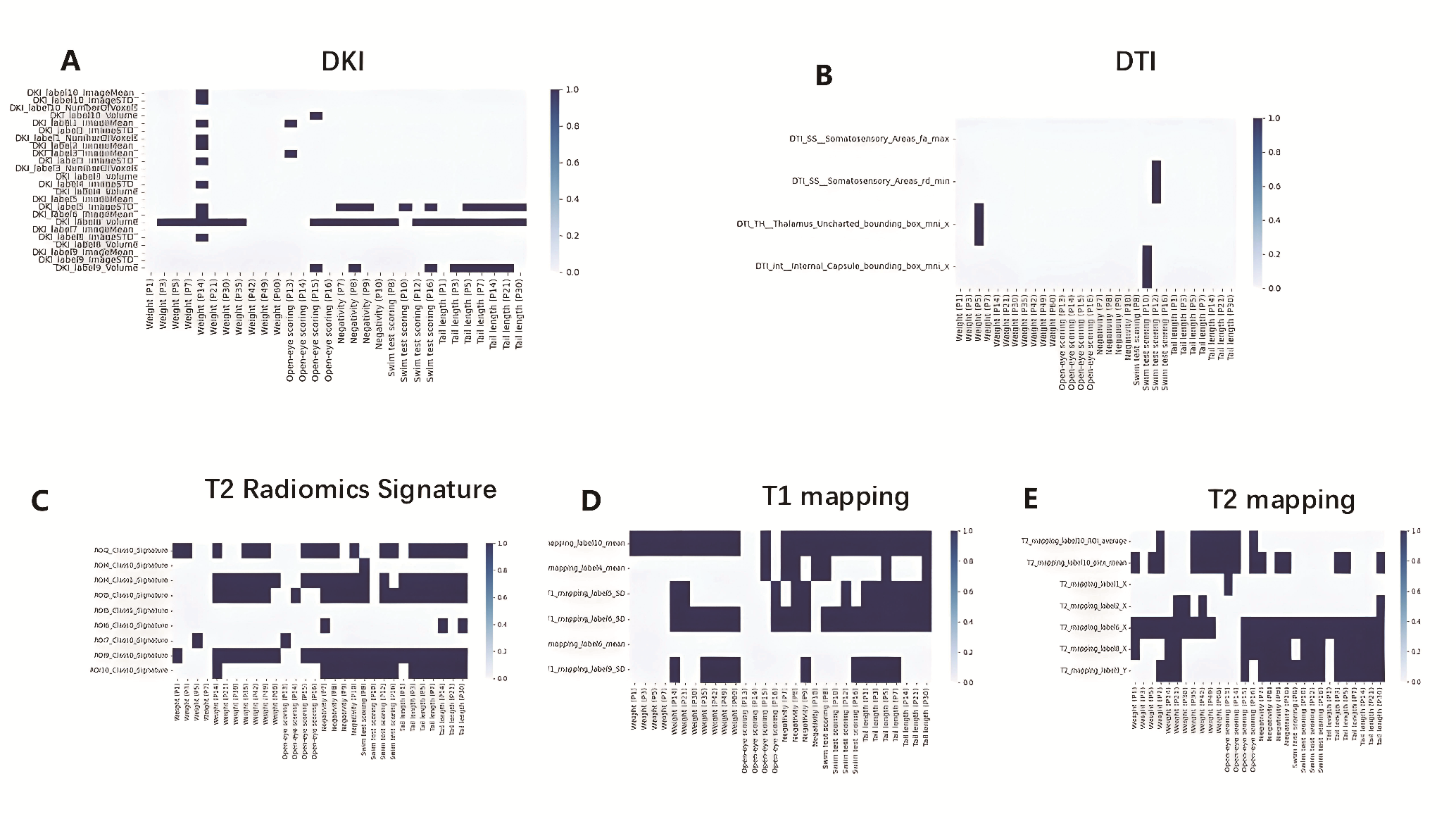
**

**Figure S16** Correlation between behavior-related measurement features and developmental characteristics: (a) in the DKI sequence; (b) in the DTI sequence; (c) in the T2 sequence; (d) in the T1 mapping sequence; (e) in the T2 mapping sequence.

**Table S1** Results of univariate logistic regression analysis. The left side presents the univariate analysis results with modeling as the prediction target, whereas the right side presents the univariate analysis results with treatment as the prediction target. (label1-10/ ROI1-10: frontal lobe; motor-somatosensory cortex; cingulate gyrus; corpus callosum; internal capsule; hippocampus; thalamus; amygdala; visual-auditory cortex; cerebellum).

|  | Model | | Treatment | |
| --- | --- | --- | --- | --- |
|  | Univariate OR (95% CI) | Univariate p-value | Univariate OR (95% CI) | Univariate p-value |
| DTI | | | | |
| DTI_SS__Somatosensory_Areas_fa_max | 9.757 (0.36-264.231) | 0.176 | 0.386 (0.015-9.77) | 0.564 |
| DTI_SS__Somatosensory_Areas_rd_min | 1.256 (0.082-19.223) | 0.870 | 3.059 (0.185-50.532) | 0.435 |
| DTI_TH__Thalamus_Uncharted_bounding_box_mni_x | 0.877 (0.412-1.869) | 0.735 | 0.808 (0.378-1.729) | 0.583 |
| DTI_int__Internal_Capsule_bounding_box_mni_x | 1.81 (0.837-3.914) | 0.132 | 0.96 (0.492-1.872) | 0.905 |
| DKI | | | | |
| DKI_label10_ImageMean_ | 1.001 (1.0-1.003) | 0.139 | 0.999 (0.997-1.001) | 0.241 |
| DKI_label10_ImageSTD_ | 1.002 (0.996-1.007) | 0.580 | 0.996 (0.99-1.002) | 0.165 |
| DKI_label10_NumberOfVoxels | 1.0 (0.998-1.001) | 0.359 | 1.0 (0.999-1.001) | 0.609 |
| DKI_label10_Volume | 0.995 (0.987-1.003) | 0.220 | 1.011 (1.002-1.021) | **0.013** |
| DKI_label1_ImageMean_ | 1.001 (0.999-1.002) | 0.354 | 0.999 (0.998-1.001) | 0.350 |
| DKI_label1_ImageSTD_ | 1.003 (0.998-1.008) | 0.269 | 0.997 (0.992-1.003) | 0.322 |
| DKI_label1_NumberOfVoxels | 1.0 (0.996-1.003) | 0.973 | 1.001 (0.997-1.004) | 0.747 |
| DKI_label2_ImageMean_ | 1.0 (0.999-1.001) | 0.590 | 1.0 (0.999-1.001) | 0.378 |
| DKI_label3_ImageMean_ | 1.002 (0.998-1.006) | 0.372 | 0.995 (0.989-1.0) | 0.064 |
| DKI_label3_ImageSTD_ | 1.0 (0.999-1.001) | 0.409 | 1.0 (0.999-1.001) | 0.914 |
| DKI_label3_NumberOfVoxels | 0.997 (0.971-1.023) | 0.822 | 0.99 (0.964-1.016) | 0.450 |
| DKI_label3_Volume | 1.0 (0.999-1.001) | 0.818 | 1.0 (0.999-1.001) | 0.480 |
| DKI_label4_ImageSTD_ | 1.0 (0.999-1.001) | 0.461 | 1.0 (0.999-1.001) | 0.713 |
| DKI_label4_Volume | 1.0 (0.999-1.001) | 0.894 | 0.999 (0.999-1.0) | 0.317 |
| DKI_label5_ImageMean_ | 1.0 (0.999-1.001) | 0.867 | 0.999 (0.998-1.0) | 0.240 |
| DKI_label5_ImageSTD_ | 1.013 (1.001-1.026) | **0.035** | 0.993 (0.981-1.004) | 0.204 |
| DKI_label6_ImageMean_ | 1.0 (0.999-1.001) | 0.623 | 0.999 (0.998-1.0) | 0.164 |
| DKI_label6_Volume | 1.049 (1.019-1.081) | **0.001** | 1.003 (0.982-1.025) | 0.769 |
| DKI_label7_ImageMean_ | 1.0 (0.999-1.001) | 0.744 | 0.999 (0.998-1.0) | 0.262 |
| DKI_label8_ImageSTD_ | 1.001 (0.999-1.002) | 0.351 | 1.0 (0.999-1.001) | 0.890 |
| DKI_label8_Volume | 1.0 (0.999-1.001) | 0.790 | 1.0 (0.998-1.001) | 0.441 |
| DKI_label9_ImageMean_ | 1.0 (0.999-1.001) | 0.653 | 1.0 (0.998-1.001) | 0.430 |
| DKI_label9_ImageSTD_ | 1.001 (0.997-1.005) | 0.723 | 0.995 (0.991-0.999) | **0.024** |
| DKI_label9_Volume | 1.007 (1.0-1.014) | **0.040** | 1.001 (0.995-1.007) | 0.768 |
| T1_mapping | | | | |
| T1_mapping_label10_mean | 0.992 (0.988-0.997) | <**0.001** | 1.0 (0.997-1.002) | 0.774 |
| T1_mapping_label4_mean | 0.998 (0.996-1.0) | **0.029** | 1.001 (0.999-1.003) | 0.255 |
| T1_mapping_label5_SD | 1.016 (1.003-1.029) | **0.017** | 0.994 (0.983-1.004) | 0.228 |
| T1_mapping_label6_SD | 1.017 (1.005-1.029) | **0.006** | 0.995 (0.988-1.002) | 0.191 |
| T1_mapping_label6_mean | 1.001 (0.999-1.003) | 0.355 | 1.001 (0.999-1.003) | 0.231 |
| T1_mapping_label9_SD | 1.008 (0.999-1.016) | 0.087 | 0.998 (0.991-1.004) | 0.439 |
| T2 mapping | | | | |
| T2_mapping_label10_ROI_average | 0.945 (0.872-1.024) | 0.168 | 0.965 (0.893-1.044) | 0.377 |
| T2_mapping_label10_piex_mean | 0.914 (0.839-0.997) | **0.042** | 0.986 (0.912-1.067) | 0.730 |
| T2_mapping_label1_X | 1.024 (0.986-1.064) | 0.220 | 0.975 (0.939-1.013) | 0.201 |
| T2_mapping_label2_X | 1.024 (0.996-1.052) | 0.093 | 0.957 (0.922-0.994) | **0.022** |
| T2_mapping_label6_X | 0.973 (0.96-0.987) | <**0.001** | 0.988 (0.976-1.001) | 0.067 |
| T2_mapping_label8_X | 0.918 (0.854-0.987) | **0.021** | 1.002 (0.961-1.045) | 0.923 |
| T2_mapping_label9_Y | 1.095 (1.024-1.17) | **0.008** | 1.104 (1.031-1.183) | **0.004** |
| T2 Radiomics Signature | | | | |
| ROI2_Class0_Signature | 0.544 (0.33-0.895) | **0.017** | 0.773 (0.496-1.206) | 0.256 |
| ROI4_Class0_Signature | 0.596 (0.243-1.459) | 0.257 | 4.656 (1.795-12.076) | **0.002** |
| ROI4_Class1_Signature | 9.691 (2.514-37.36) | **0.001** | 0.143 (0.042-0.482) | **0.002** |
| ROI5_Class0_Signature | 0.057 (0.006-0.514) | **0.011** | 1.407 (0.404-4.898) | 0.592 |
| ROI5_Class1_Signature | 1.438 (0.568-3.643) | 0.444 | 2.606 (1.007-6.744) | **0.048** |
| ROI7_Class0_Signature | 0.724 (0.462-1.135) | 0.160 | 0.831 (0.536-1.288) | 0.408 |
| ROI6_Class0_Signature | 2.622 (0.927-7.418) | 0.069 | 0.552 (0.198-1.539) | 0.256 |
| ROI9_Class0_Signature | 0.122 (0.039-0.387) | <**0.001** | 1.669 (0.614-4.538) | 0.316 |
| ROI10_Class0_Signature | 0.093 (0.018-0.495) | **0.005** | 0.64 (0.298-1.376) | 0.253 |

**Table S2** Results of multivariate logistic regression analysis with modeling as the prediction target. The left two columns show the results of individual unimodal models, whereas the right two columns show the results of a combined multimodal model. (label1-10/ ROI1-10: frontal lobe; motor-somatosensory cortex; cingulate gyrus; corpus callosum; internal capsule; hippocampus; thalamus; amygdala; visual-auditory cortex; cerebellum)

|  | Single modality radscore | | Multi-modality radscore | |
| --- | --- | --- | --- | --- |
|  | Multivariate OR (95% CI) | Multivariate p-value | Multivariate OR (95% CI) | Multivariate p-value |
| DTI | | | | |
| DTI_int__Internal_Capsule_bounding_box_mni_x | 1.81 (0.837-3.914) | 0.132 |  |  |
| DKI | | | | |
| DKI_label5_ImageSTD_ | 1.012 (0.998-1.026) | 0.099 | 1.025 (0.983-1.069) | 0.242 |
| DKI_label6_Volume | 1.047 (1.017-1.078) | **0.002** | 1.055 (0.975-1.142) | 0.183 |
| DKI_label9_Volume | 1.003 (0.995-1.011) | 0.504 | 1.002 (0.982-1.023) | 0.820 |
| T1 mapping | | | | |
| T1_mapping_label10_mean | 0.99 (0.984-0.995) | <**0.001** | 0.982 (0.966-0.998) | **0.028** |
| T1_mapping_label4_mean | 0.998 (0.995-1.001) | 0.123 | 1.004 (0.993-1.015) | 0.444 |
| T1_mapping_label5_SD | 1.033 (1.007-1.06) | **0.013** | 1.026 (0.969-1.087) | 0.370 |
| T1_mapping_label6_SD | 1.017 (1.0-1.035) | **0.044** | 1.037 (0.998-1.078) | 0.067 |
| T2 mapping | | | | |
| T2_mapping_label10_piex_mean | 0.899 (0.81-0.998) | **0.045** | 0.746 (0.529-1.051) | 0.094 |
| T2_mapping_label6_X | 0.98 (0.964-0.995) | **0.011** | 0.955 (0.902-1.012) | 0.117 |
| T2_mapping_label8_X | 0.947 (0.887-1.012) | 0.107 | 0.918 (0.763-1.105) | 0.368 |
| T2_mapping_label9_Y | 1.072 (0.986-1.165) | 0.104 | 1.208 (0.933-1.566) | 0.152 |
| T2 Radiomics Signature | | | | |
| ROI2_Class0_Signature | 0.958 (0.502-1.828) | 0.896 | 6.068 (0.292-125.935) | 0.244 |
| ROI4_Class1_Signature | 7.812 (1.496-40.801) | **0.015** | 719.792 (0.718-721406.0) | 0.062 |
| ROI5_Class0_Signature | 0.124 (0.012-1.238) | 0.075 | 0.04 (0.0-5.729) | 0.203 |
| ROI9_Class0_Signature | 0.446 (0.106-1.875) | 0.270 | 0.361 (0.003 - 42.534) | 0.676 |
| ROI10_Class0_Signature | 0.117 (0.013-1.068) | 0.057 | 0.002 (0.0-23.973) | 0.193 |

**Table S3** Results of multivariate logistic regression analysis with treatment as the prediction target. The left two columns show the results of individual unimodal models, whereas the right two columns show the results of a combined multimodal model. (label1-10/ ROI1-10: frontal lobe; motor-somatosensory cortex; cingulate gyrus; corpus callosum; internal capsule; hippocampus; thalamus; amygdala; visual-auditory cortex; cerebellum)

|  | Single modality radscore | | Multi-modality radscore | |
| --- | --- | --- | --- | --- |
|  | Multivariate OR (95% CI) | Multivariate p-value | Multivariate OR (95% CI) | Multivariate p-value |
| DTI | | | | |
| DTI_SS__Somatosensory_Areas_rd_min | 3.059 (0.185-50.532) | 0.435 |  |  |
| DKI | | | | |
| DKI_label10_Volume | 1.015 (1.005-1.025) | **0.004** | 1.008 (0.995-1.022) | 0.212 |
| DKI_label9_ImageSTD_ | 0.993 (0.988-0.998) | **0.007** | 0.987 (0.978-0.996) | **0.004** |
| T1 mapping | | | | |
| T1_mapping_label6_SD | 0.995 (0.988-1.002) | 0.191 |  |  |
| T2 mapping | | | | |
| T2_mapping_label9_Y | 1.1 (1.023-1.184) | **0.010** | 1.286 (1.107-1.493) | **0.001** |
| T2_mapping_label2_X | 0.963 (0.931-0.997) | **0.033** | 0.965 (0.924-1.009) | 0.121 |
| T2 Radiomics Signature | | | | |
| ROI4_Class0_Signature | 3.97 (1.013-15.556) | **0.048** | 4.456 (0.548-36.204) | 0.162 |
| ROI4_Class1_Signature | 0.308 (0.065-1.458) | 0.138 | 0.227 (0.022-2.38) | 0.216 |
| ROI5_Class1_Signature | 4.613 (1.422-14.966) | **0.011** | 4.179 (0.944-18.505) | 0.06 |

**Table S4** Accuracy, sensitivity, and specificity of unimodal and multimodal imaging scoring.

|  | Model | | | Treatment | | |
| --- | --- | --- | --- | --- | --- | --- |
|  | Accuracy | Sensitivity | Specificity | Accuracy | Sensitivity | Specificity |
| DKI | 0.719 | 0.714 | 0.725 | 0.744 | 0.738 | 0.75 |
| DTI | 0.631 | 0.597 | 0.643 | 0.551 | 0.488 | 0.619 |
| T2 Radiomics Signature | 0.695 | 0.714 | 0.675 | 0.707 | 0.881 | 0.525 |
| T1 mapping | 0.780 | 0.762 | 0.800 | 0.524 | 0.738 | 0.300 |
| T2 mapping | 0.720 | 0.762 | 0.675 | 0.671 | 0.738 | 0.600 |
| Multi-modality | 0.927 | 0.929 | 0.925 | 0.805 | 0.810 | 0.800 |

**Table S5** Correlation analysis of two multimodal imaging scores with behavioral results. The Pearson correlation coefficient (r) and its p-value were calculated, and the results are shown in the table below.

|  |  | Stats | p-value |
| --- | --- | --- | --- |
| Model RadScore | Percentage of dwell time | -0.466 | <0.001 |
|  | Social time (phase 1, Stranger 1) | -0.405 | <0.001 |
|  | Social time (phase 2, Stranger 2) | -0.250 | 0.024 |
|  | Percentage of new object learning | -0.513 | <0.001 |
|  | Number of beads buried | 0.501 | <0.001 |
| Treatment RadScore | Percentage of dwell time | 0.232 | 0.036 |
|  | Social time (phase 1, empty) | -0.299 | 0.006 |
|  | Percentage of new object learning | 0.375 | <0.001 |
|  | Number of beads buried | -0.429 | <0.001 |

**Table S6** Correlation analysis of two multimodal imaging scores with developmental characteristics. For continuous developmental traits, Pearson correlation coefficients (r) and their p-values were calculated. For discrete developmental traits (Open-eye scoring, Swim test scoring), one-way ANOVA F-statistics and their p-values were calculated. The results are shown in the table below.

|  |  | Stats | p-value |
| --- | --- | --- | --- |
| Model RadScore | Weight (P1) | -0.528 | <0.001 |
| Model RadScore | Weight (P3) | -0.537 | <0.001 |
| Model RadScore | Weight (P5) | -0.526 | <0.001 |
| Model RadScore | Weight (P7) | -0.618 | <0.001 |
| Model RadScore | Weight (P14) | -0.726 | <0.001 |
| Model RadScore | Weight (P21) | -0.763 | <0.001 |
| Model RadScore | Weight (P30) | -0.748 | <0.001 |
| Model RadScore | Weight (P35) | -0.765 | <0.001 |
| Model RadScore | Weight (P42) | -0.725 | <0.001 |
| Model RadScore | Weight (P49) | -0.657 | <0.001 |
| Model RadScore | Weight (P60) | -0.446 | <0.001 |
| Model RadScore | Open-eye scoring (P13) | 6.058 | 0.003 |
| Model RadScore | Open-eye scoring (P14) | 9.709 | <0.001 |
| Model RadScore | Open-eye scoring (P15) | 45.583 | <0.001 |
| Model RadScore | Open-eye scoring (P16) | 27.192 | <0.001 |
| Model RadScore | Negativity (P7) | 0.821 | <0.001 |
| Model RadScore | Negativity (P8) | 0.859 | <0.001 |
| Model RadScore | Negativity (P9) | 0.877 | <0.001 |
| Model RadScore | Negativity (P10) | 0.761 | <0.001 |
| Model RadScore | Swim test scoring (P8) | 124.264 | <0.001 |
| Model RadScore | Swim test scoring (P10) | 31.104 | <0.001 |
| Model RadScore | Swim test scoring (P12) | 189.323 | <0.001 |
| Model RadScore | Swim test scoring (P16) | 64.510 | <0.001 |
| Model RadScore | Tail length (P1) | -0.725 | <0.001 |
| Model RadScore | Tail length (P3) | -0.821 | <0.001 |
| Model RadScore | Tail length (P5) | -0.832 | <0.001 |
| Model RadScore | Tail length (P7) | -0.848 | <0.001 |
| Model RadScore | Tail length (P14) | -0.855 | <0.001 |
| Model RadScore | Tail length (P21) | -0.892 | <0.001 |
| Model RadScore | Tail length (P30) | -0.905 | <0.001 |

Scan parameters:

(1) T2-weighted imaging was performed with the following parameters: TE/TR=51/5000, FOV=30x30 mm, Matrix=256×238, number of layers=20, layer thickness=1 mm. Scan time = 5 min and 30 s.

(2) T1 mapping was performed with the following parameters: repetition time (TR) = 20, echo time (TE) = 0, slices = 32, thickness = 0.75, slice gap = 1, FOV=30*24, matrix = 160*120, scanning time 12 min and 48 s.

(3) T2 mapping was performed with the following parameters: repetition time (TR) = 1500, echo time (TE) = 13, slices = 16, thickness = 1, slice gap = 1, FOV = 30 * 30, scan matrix = 192 * 128, scan time 9 min 36 s.

(4) DTI was performed with the following parameters: repetition time (TR) = 5000, echo time (TE) = 27, slices = 22, thickness = 1, slice gap = 1, FOV = 22 * 22, scan matrix = 100 * 114, scan time = 22 min and 30 s.

(5) DKI was performed using Echo Planar Imaging (EPI) sequences: 3 b values (b=0 s/mm2, 1000 s/mm2, 2000 s/mm2) and 30 diffusion coding directions. All rats use the same gradient table when performing DKI scans. The scanning method is to scan 5 b0 images consecutively, then b=1000 s/mm2, and 2000 s/mm2 alternately scanned. DKI scan parameters: TR/TE=4000/22 ms; matrix = 46×44 mm; FOV =26×22.75 mm；Layer thickness = 1 mm. The duration of the scan is approximately 59 min.
